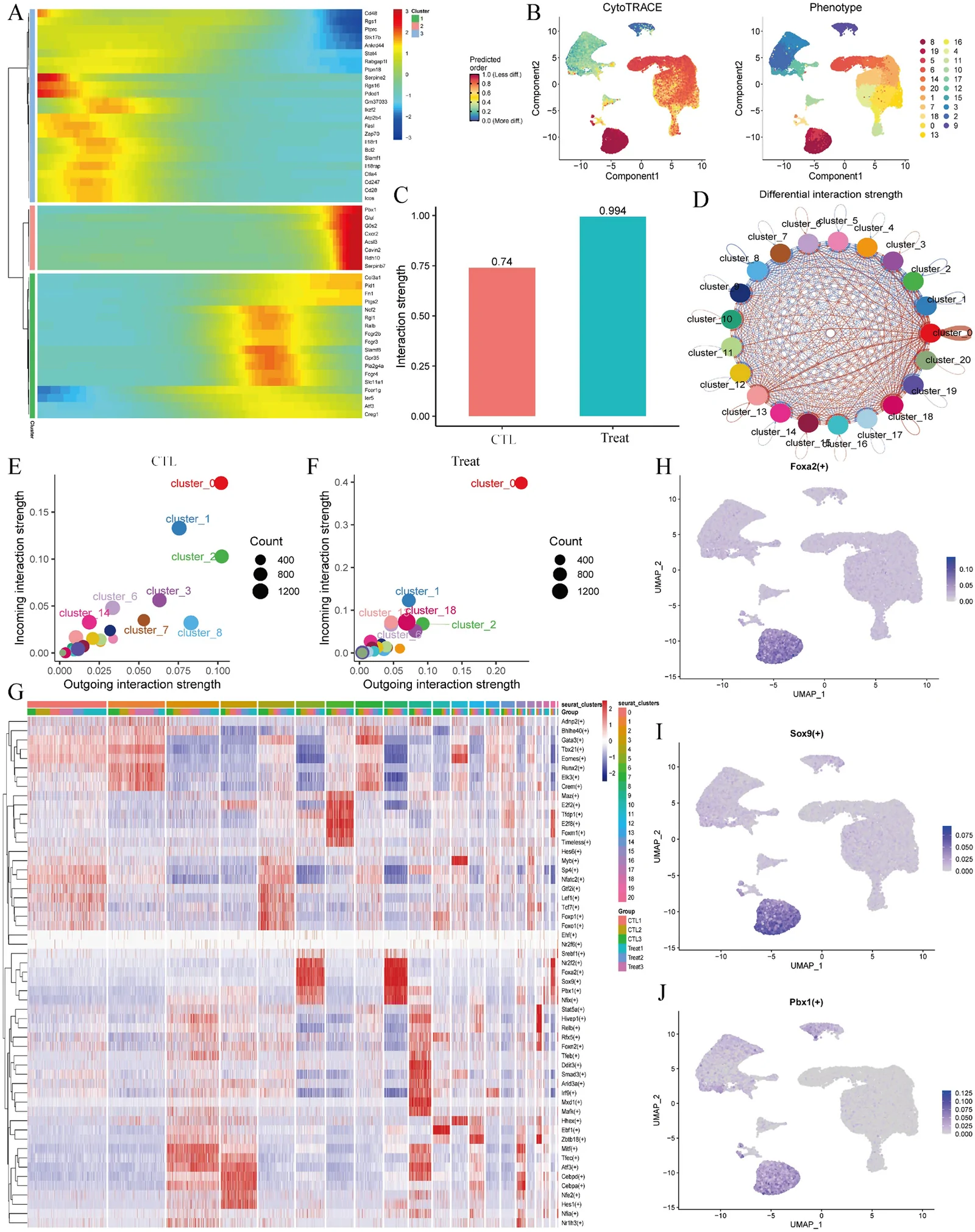# A high-affinity anti-ITGB5 nanobody for hepatocellular carcinoma: Antitumor efficacy and tumor microenvironment reprogramming

**DOI:** 10.1016/j.jbc.2026.113423

**Published:** 2026-08-11

**Authors:** Wenzhang Wu, Daijun Wang, Caihao Qu, Yumin Li

**Affiliations:** 1 Department of Surgical Oncology, The Second Hospital of Lanzhou University (Second Clinical Medical College), Lanzhou City, Gansu Province, China; 2 Gansu Province Key Laboratory of Environmental Oncology, Lanzhou City, Gansu Province, China; 3 Department of General Surgery, The Second Hospital of Lanzhou University (Second Clinical Medical College), Lanzhou University, Lanzhou City, Gansu Province, China

**Keywords:** hepatocellular carcinoma, ITGB5, nanobody, single-cell RNA sequencing, target therapy

## Abstract

Although immune checkpoint blockade has improved systemic therapy for hepatocellular carcinoma (HCC), durable benefit is limited by primary resistance, poor tumor penetration of antibodies, and an immunosuppressive tumor microenvironment. We identified integrin β5 (ITGB5) as an oncogenic and immune-associated target in HCC and developed an ITGB5-directed nanobody. Integrative analyses of TCGA/ICGC/TIMER2.0/Human Protein Atlas datasets, tissue microarrays, and functional assays showed that ITGB5 is upregulated in HCC and correlates with poor prognosis, higher tumor grade, increased immunosuppressive macrophage infiltration, reduced CD8+ T-cell abundance, and predicted resistance to sorafenib, oxaliplatin, and axitinib. Gain- and loss-of-function studies demonstrated that ITGB5 promotes proliferation, clonogenicity, migration/invasion, cell-cycle progression, and tumor growth while suppressing apoptosis, with transcriptomic signatures implicating extracellular matrix–receptor interaction, cytokine signaling, TNF/NF-κB, MAPK, and TGF-β pathways. From a yeast-display library we isolated AntiITGB5-Nb#2, a high-affinity nanobody that recognizes human and murine ITGB5, validated by flow cytometry, immunofluorescence, molecular docking, and surface plasmon resonance. AntiITGB5-Nb#2 inhibited growth and motility of ITGB5-high HCC cells and suppressed tumor progression in subcutaneous and orthotopic models, including an immunocompetent murine HCC model, without overt toxicity. Single-cell RNA sequencing revealed tumor microenvironment remodeling characterized by enhanced cytotoxic immune infiltration and reduced immunosuppressive signaling. These results establish ITGB5 as a prognostic, therapeutically actionable target in HCC and support AntiITGB5-Nb#2 as a nanobody-based strategy to complement current therapies.

Hepatocellular carcinoma (HCC) is the dominant histological subtype of primary liver cancer and remains a major cause of cancer-related mortality worldwide. Despite advances in surveillance, surgical resection, liver transplantation, locoregional therapy and systemic treatment, the prognosis of patients with advanced HCC remains unsatisfactory (1). In 2022, liver cancer accounted for more than 865,000 new cases and 757,000 deaths globally, ranking among the leading causes of cancer mortality (2). The disease burden is expected to continue increasing, underscoring the need for more effective and mechanistically informed therapeutic strategies.

Systemic therapy for HCC has evolved rapidly over the past decade (3, 4). Sorafenib and lenvatinib were the first tyrosine kinase inhibitors approved as first-line options for advanced disease (5, 6). More recently, immune checkpoint inhibitors (ICIs), either alone or in combination with antiangiogenic agents or multi-kinase inhibitors, have substantially improved clinical outcomes. Regimens targeting the PD-1/PD-L1 and CTLA-4 axes have extended median survival in selected patients and are now central components of first-line treatment (7, 8, 9). Nevertheless, several major limitations remain. First, reliable biomarkers for predicting response, resistance and prognosis are lacking. Second, a considerable proportion of patients exhibit primary resistance or acquire resistance after initial benefit. Third, conventional monoclonal antibodies, because of their large molecular size and limited tumor penetration, often show suboptimal accumulation in solid tumors with dense stromal barriers. Finally, excessive therapeutic dependence on VEGF- and PD-1/PD-L1-centered pathways may fail to overcome the complex immunosuppressive architecture of the HCC microenvironment.

These challenges highlight the importance of identifying novel actionable targets and developing next-generation biologics with improved tissue accessibility and therapeutic versatility. Conventional full-length monoclonal antibodies consist of paired heavy and light chains as well as an Fc fragment. In contrast, nanobodies are isolated VHH variable domains derived from camelid heavy-chain-only antibodies (∼15 kDa), representing the smallest naturally occurring antigen-binding fragments without light chains or constant Fc regions. This distinct structural composition serves as the fundamental criterion differentiating nanobodies from conventional antibodies.Compared with full-length antibodies; nanobodies exhibit prominent strengths including high stability, superior tumor tissue penetration, low immunogenicity, convenient genetic modification and low manufacturing cost (10). Such merits render them ideal tools for solid tumor intervention, functioning as direct blocking agents, molecular imaging tracers, immune regulators or carriers for cytotoxic drugs (11, 12).

Identified integrin β5 (ITGB5) is a member of the integrin β subfamily and forms the αvβ5 heterodimer with the αv subunit. Integrins act as bidirectional signaling receptors that connect extracellular matrix (ECM) cues to intracellular oncogenic programs controlling cell adhesion, motility, survival and immune communication (13). The extracellular domain of ITGB5 contains a PSI domain, hybrid domain, βI domain and cysteine-rich EGF-like repeats (14), which collectively mediate ligand recognition, divalent cation-dependent conformational switching and downstream signal transductions (15). Previous studies have implicated ITGB5 in tumor proliferation, invasion, metastasis and drug resistance across multiple cancer types (16). In HCC, ITGB5 has been linked to activation of AKT-mTOR signaling and reduced sorafenib sensitivity, suggesting that it may coordinate malignant phenotypes and therapeutic resistance (17). Accumulating evidence indicates that ITGB5 exerts critical oncogenic functions in tumor progression. Based on the above findings, we propose the core research hypothesis of this study: ITGB5 is aberrantly upregulated in HCC, drives the malignant progression of hepatocellular carcinoma, and can serve as a novel prognostic biomarker and therapeutic target for HCC.

However, whether ITGB5 can be exploited as a nanobody-targetable immunotherapeutic node in HCC remains unclear. In this study, we systematically evaluated the expression, prognostic value, biological function and immune relevance of ITGB5 in HCC. We then generated a specific ITGB5-targeting nanobody using yeast-display technology and assessed its binding characteristics, antitumor efficacy and capacity to remodel the immune microenvironment. Our findings identify ITGB5 as a clinically relevant oncogenic driver and establish AntiITGB5-Nb#2 as a promising nanobody candidate for translational HCC therapy.

## Results

### *ITGB5* is upregulated in HCC and predicts poor prognosis

**Figure 1:**
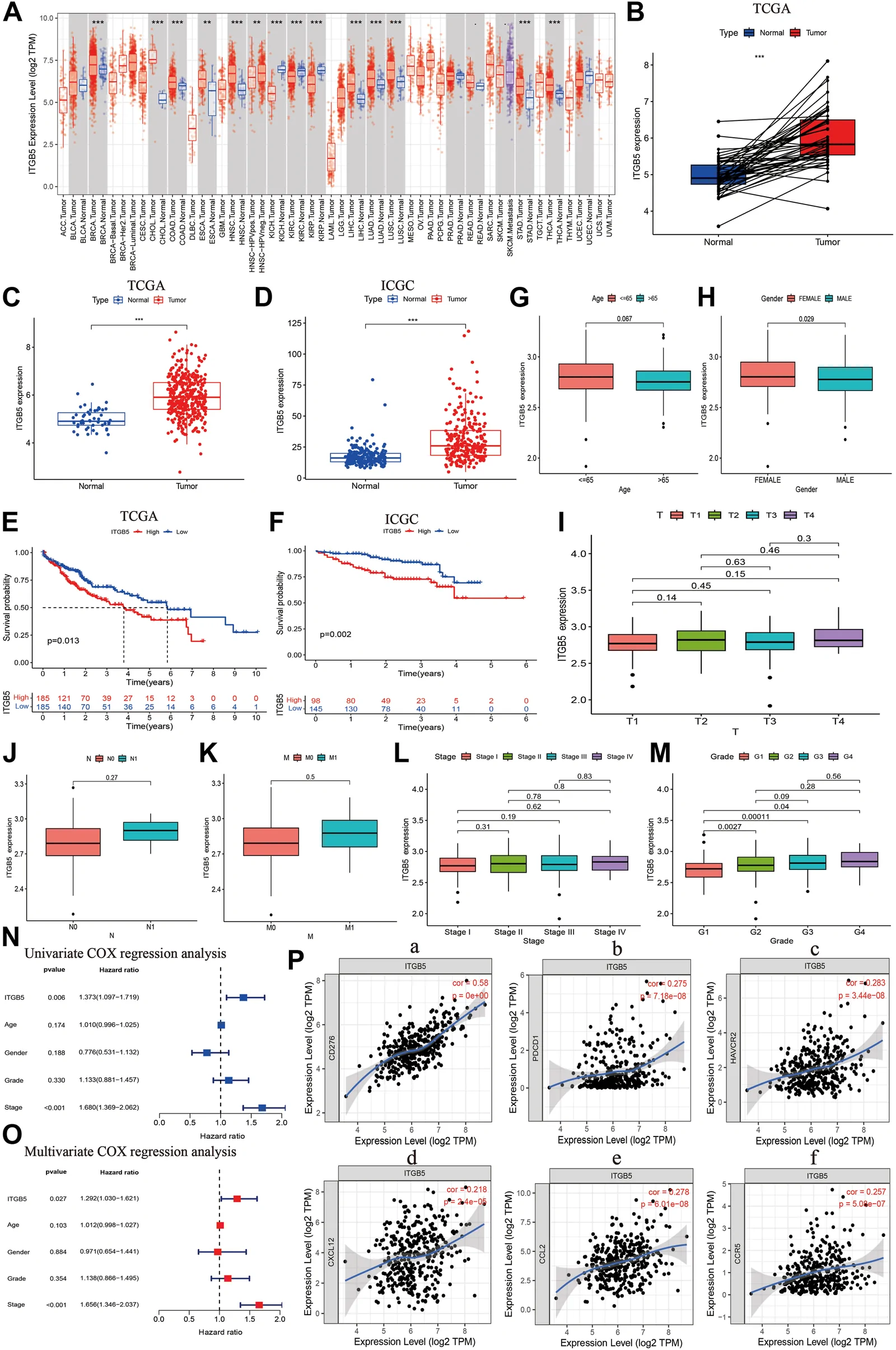
**Upregulated *ITGB5* in HCC correlates with unfavorable clinicopathological characteristics, poor clinical prognosis and multiple immune regulatory molecules.***A*, pan-cancer expression landscape of *ITGB5* across all tumor types in the TCGA dataset; *B* and *C*, differential expression of *ITGB5* between HCC tumor and normal liver tissues in the TCGA cohort: *B*, paired tumor-adjacent samples and (*C*) unpaired independent samples; *D*, verification of *ITGB5* upregulation in HCC *versus* matched peritumoral normal tissues based on the ICGC database; *E* and *F*, Kaplan–Meier survival curves comparing overall survival between patients with high and low *ITGB5* expression in the TCGA (*E*) and ICGC (*F*) HCC cohorts; *G and H*, correlation analysis between *ITGB5* expression and clinical demographic indicators including patient age (*G*) and gender (*H*) from TCGA; *I and M*, associations of *ITGB5* expression with tumor pathological parameters: T classification (*I*), N classification (*J*), M classification (*K*), overall clinical stage (*L*), and pathological differentiation grade *(M*); *N and O*, univariate (*N*) and multivariate (*O*) Cox proportional hazards regression analyses to identify independent prognostic risk factors for HCC; *P*, scatter correlation plots showing the linear associations between ITGB5 expression and representative immune regulatory genes, including *CD276*, *PDCD1*, *HAVCR2*, *CXCL12*, *CCL2* and *CCR5* (subgraphs a–f respectively). All quantitative data are presented as mean ± SD. Statistical significance was determined by corresponding bioinformatic statistical tests, ∗*p* < 0.05, ∗∗*p* < 0.01, ∗∗∗*p* < 0.001. HCC, hepatocellular carcinoma; ITGB5, identified integrin β5.

Pan-cancer analysis of TCGA datasets revealed that *ITGB5* was significantly overexpressed in multiple malignancies, including liver hepatocellular carcinoma (Fig. 1*A*). Both paired (Fig. 1*B*) and unpaired(Fig. 1*C*) analyses of TCGA-LIHC samples, as well as validation in the ICGC cohort(Fig. 1*D*), confirmed that *ITGB5* mRNA expression was markedly higher in HCC tissues than in adjacent non-tumor liver tissues. Kaplan-Meier analysis demonstrated that patients with high *ITGB5* expression had significantly shorter overall survival in both TCGA (Fig. 1*E*) and ICGC (Fig. 1*F*) cohorts, suggesting that *ITGB5* overexpression is tightly correlated with poor clinical prognosis of HCC patients.

**Fig. S1:**
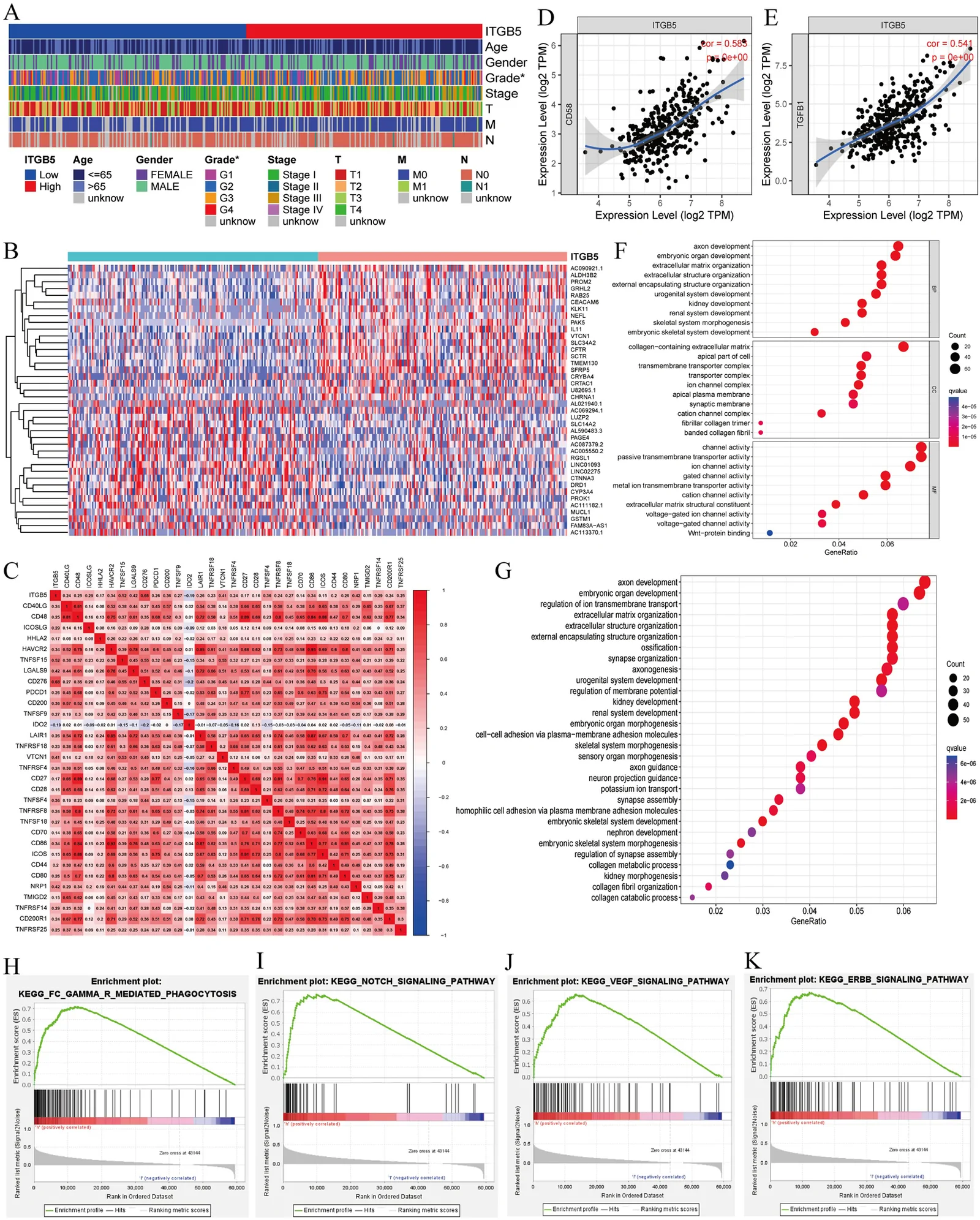


Subsequently, we analyzed the correlation between *ITGB5* expression and diverse clinical pathological features in the TCGA-LIHC cohort, including patient age, gender, T stage, N stage, M stage, clinical stage, and pathological grade (Fig. 1, *G*–M). Differential analysis showed that *ITGB5* expression was significantly higher in female HCC patients (*p* = 0.029) (Fig. 1*H*). Notably, *ITGB5* expression increased gradually along with the elevation of pathological grade (*p* < 0.05) (Figs. 1*M*, S1*A*), strongly indicating that aberrant *ITGB5* upregulation is closely coupled with the progressive malignant deterioration of HCC. Furthermore, univariate Cox regression analysis (Fig. 1*N*) and multivariate Cox regression analysis (Fig. 1*O*) revealed that high *ITGB5* expression was an independent risk factor affecting patient overall survival or recurrence risk (*p* < 0.05, HR > 1), further supporting the clinical prognostic value of ITGB5 in HCC.

To preliminarily explore the potential biological mechanism and immune regulatory role of ITGB5 in HCC progression, we performed correlation analysis of key HCC malignant molecules and immune regulatory genes. Heatmap analysis revealed that *ITGB5* expression was positively correlated with multiple oncogenic molecules driving HCC progression, such as *TMEM130* and *CEACAM6* (Fig. S1*B*), suggesting that *ITGB5* may cooperate with protumor genes to facilitate HCC malignant progression. More importantly, *ITGB5* expression was positively correlated with a panel of critical immune checkpoint molecules, including *TNFSF15*, *CD200*, *VTCN1*, *TNFSF4*, and *NRP1* (Fig. S1*C*). Consistently, TIMER2.0 database analysis further validated that *ITGB5* levels were positively correlated with multiple immune suppressive molecules and tumor microenvironment regulators, including *CD276* (Fig. 1*P*(*a*)), *PDCD1* (Fig. 1*P*(*b*)), *HAVCR2* (Fig. 1*P*(*c*)), *CXCL12* (Fig. 1*P*(*d*)), *CCL2* (Fig. 1*P*(*e*)), *CCR5* (Fig. 1*P*(*f*)), *CD58* (Fig. S1*D*), and *TGFB1* (Fig. S1*E*) (*p* < 0.0001, cor>0.2). Collectively, these bioinformatics results systematically demonstrate that elevated *ITGB5* expression is a robust malignant signature of HCC, which not only correlates with advanced pathological phenotypes and poor clinical outcomes, but also closely participates in remodeling an immunosuppressive TME. These findings strongly support our core hypothesis that ITGB5 acts as a critical oncogenic driver and promising prognostic biomarker as well as a potential immunotherapeutic target in HCC.

### *ITGB5* is linked to oncogenic signaling, immune suppression and drug resistance

Gene Ontology (GO) enrichment analysis indicated that *ITGB5*-associated Differentially expressed gene (DEGs) were primarily enriched in extracellular matrix organization, ECM structural components, ion channel complexes, and transmembrane transport functions (Fig. S1*F*). As a key ECM adhesion molecule, ITGB5-mediated regulation of ECM remodeling provides a fundamental structural basis for HCC cell invasion, migration, and malignant phenotypic transformation, revealing its intrinsic functional linkage with tumor stroma alteration.

**Figure 2:**
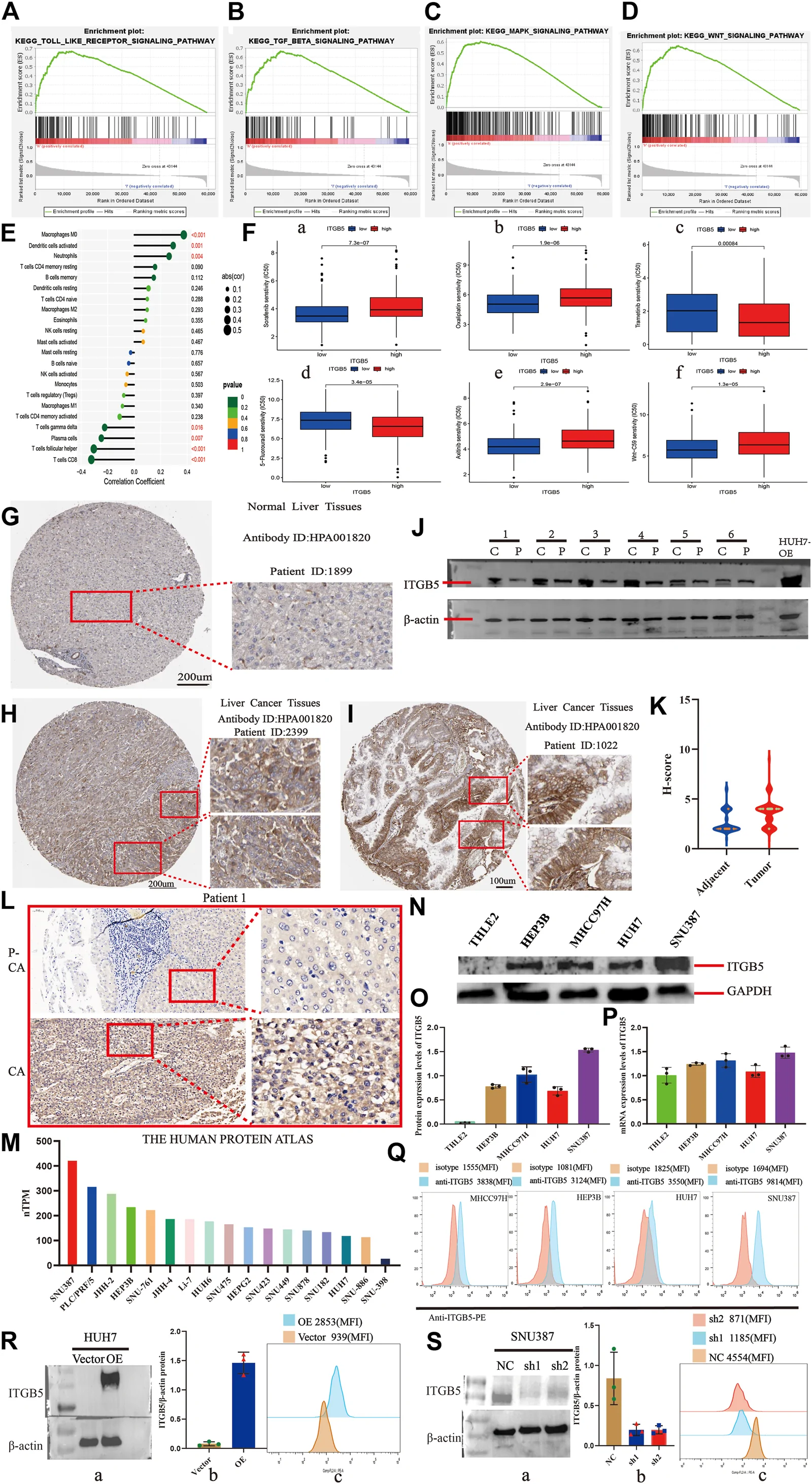
**ITGB5 is associated with oncogenic signaling, immune infiltration and therapeutic resistance, and is validated as a membrane-enriched protein in HCC tissues and cell lines.***A* and *D*, GSEA enrichment analysis of *ITGB5*-associated DEGs; *E*, correlation analysis evaluating the relationship between ITGB5 expression level and the infiltration abundance of various tumor immune cells; *F*, bioinformatic prediction of the correlation between ITGB5 expression and chemotherapeutic drug sensitivity; *G* and *I* immunohistochemical results from the HPA database showing ITGB5 expression in normal liver tissues (*G*) and hepatocellular carcinoma tissues (*H and I*); *J*, WB results of ITGB5 in six paired tumor (*C*) and adjacent normal (*P*) tissues from HCC patients; *K*, statistical scoring of immunohistochemical staining for ITGB5 in 140 paired HCC and adjacent normal tissue microarrays; *L*, immunohistochemical staining of tissue microarrays showing high expression of ITGB5 in HCC tissues, mainly localized to the cell membrane; *M*, TPM expression levels of ITGB5 in different liver cancer cell lines from the HPA database; *N* and *P*,WB (*N*), protein quantification (*O*), and RT-qPCR (*P*) results of ITGB5 in five cell lines; *Q*, validation of ITGB5 expression levels in different cell lines by flow cytometry; *R*, validation of infection efficiency of ITGB5 overexpression lentivirus in HUH7 cells by WB (*a* and *b*) and flow cytometry (*c*); *S*, validation of infection efficiency of ITGB5 knockdown lentivirus in SNU387 cells by WB (*a* and *b*) and flow cytometry (*c*). Data are expressed as mean ± SD. DEG, differentially expressed gene; GSEA, gene set enrichment analysis; HCC, hepatocellular carcinoma; ITGB5, identified integrin β5.

Kyoto Encyclopedia of Genes and Genome (KEGG) (Fig. S1*G*) and gene set enrichment analysis (GSEA) analyses further identified enrichment of multiple tumor-associated pathways, including Toll-like receptor (Fig. 2*A*),TGF-β(Fig. 2*B*), MAPK (Fig. 2*C*), WNT(Fig. 2*D*), Fcγ receptor-mediated phagocytosis (Fig. S1*H*), Notch (Fig. S1*I*), VEGF(Fig. S1*J*) and ERBB(Fig. S1*K*) pathways. Collectively, these canonical tumor-proliferative, metastatic, and immune regulatory pathways strongly illustrate that ITGB5 may participate in multi-dimensional regulation of HCC malignant behaviors, including cell proliferation, epithelial-mesenchymal transition, angiogenesis, and immune microenvironment remodeling, further explaining the poor clinical prognosis of ITGB5-high HCC patients.

Immune infiltration analysis revealed that *ITGB5* expression was positively correlated with macrophage infiltration, while negatively correlated with CD8^+^T cells and γδ T cells. This pattern suggests that ITGB5 may contribute to immune evasion by promoting immunosuppressive myeloid infiltration and limiting effector lymphocyte accumulation. (Fig. 2*E*).

Drug sensitivity analysis indicated that tumors with high ITGB5 expression had increased predicted IC_50_ values for sorafenib (Fig. 2*F*(*a*)), oxaliplatin (Fig. 2*F*(*b*)), axitinib (Fig. 2*F*(*e*)) and Wnt-C59(Fig. 2*F*(*f*)), implying reduced sensitivity to these agents. In contrast, high ITGB5 expression was associated with lower predicted IC_50_ values for trametinib (Fig. 2*F*(*c*)) and 5-fluorouracil (Fig. 2*F*(*d*)). These results demonstrate that ITGB5 expression levels are closely linked to heterogeneous drug responses in HCC patients, verifying that ITGB5 can serve as a novel biomarker for clinical therapeutic stratification, drug resistance prediction, and personalized treatment decision-making for HCC.

### ITGB5 is highly expressed on the membrane of HCC tissues and cells

**Fig. S2:**
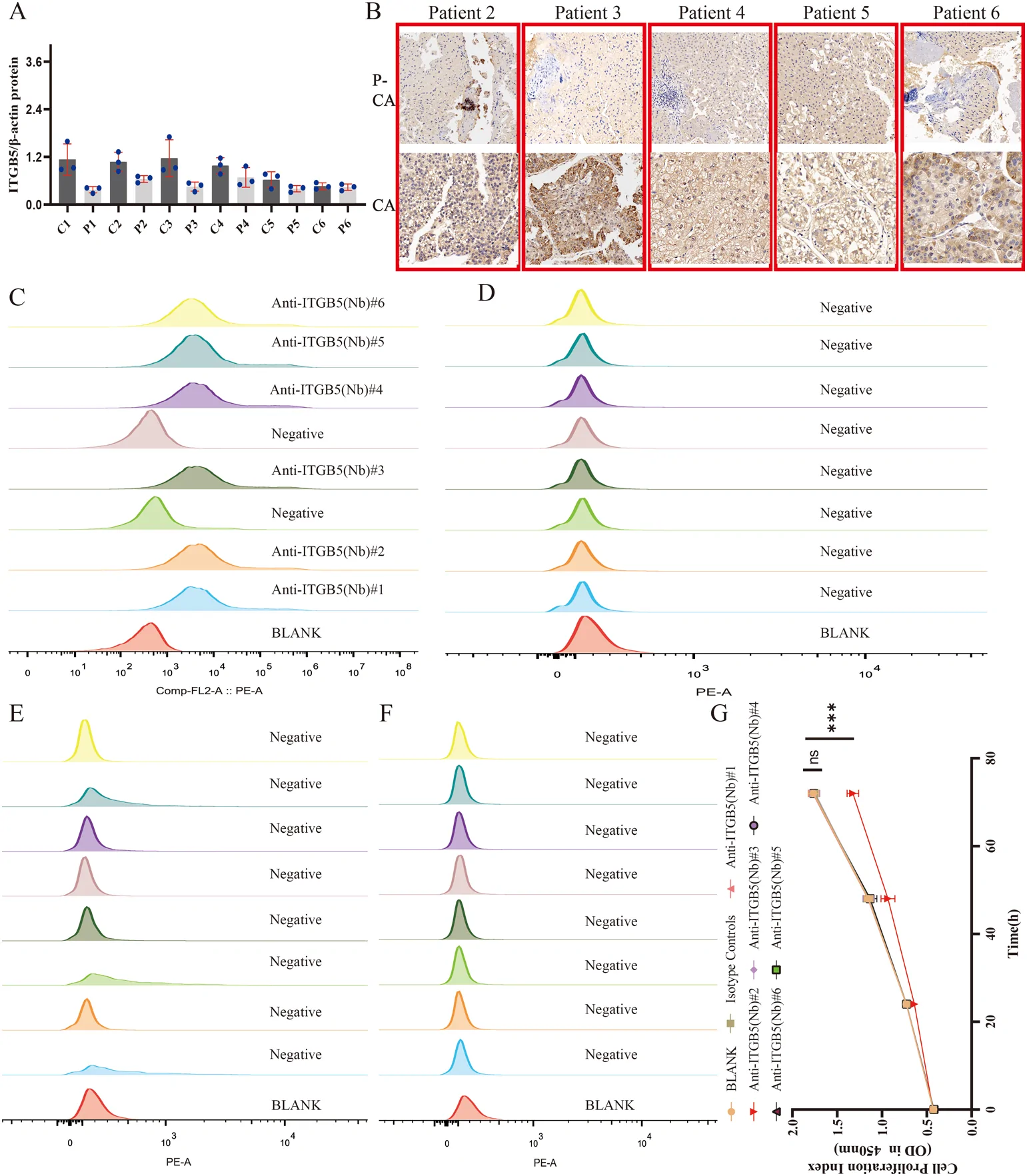


Immunohistochemical analysis based on the HPA database showed low expression of ITGB5 in normal liver tissues (Fig. 2*G*), whereas its expression was significantly elevated in HCC tissues with strong positive staining, mainly localized to the cell membrane (Fig. 2, *H* and I). Consistently, tissue microarray analysis of 140 paired HCC specimens revealed that ITGB5 was significantly upregulated in tumors compared with adjacent liver tissues. Using an H-score ≥ 3 as the cutoff, 67.9% of HCC tissues were ITGB5-positive, and 10.7% showed strong positivity (Figs. 2, *L* and *K*, S2*B*). The predominant membrane localization supports the feasibility of antibody- or nanobody-mediated targeting.

Western blotting of six paired fresh HCC samples confirmed higher ITGB5 protein expression in tumors than in adjacent tissues (Figs. 2*J*, S2*A*). Among liver cell lines, ITGB5 expression was observed in the HPA database (Fig. 2*M*). SNU387 and MHCC97H cells exhibited relatively high ITGB5 expression, whereas HUH7 cells showed low endogenous expression. These patterns were validated by qRT-PCR(Fig. 2*P*), western blotting (Fig. 2, *N* and *O*) and flow cytometry (Fig. 2*Q*). Accordingly, ITGB5-overexpressing HUH7 cells (WB (Fig. 2*R*(*a–b*)) and flow cytometry (Fig. 2*R*(*c*))) and ITGB5-knockdown SNU387 cells(WB(Fig. S2(*a–b*)) and flow cytometry (Fig. S2(*c*)))were generated for subsequent functional studies.

### ITGB5 promotes malignant phenotypes of HCC cells

**Figure 3:**
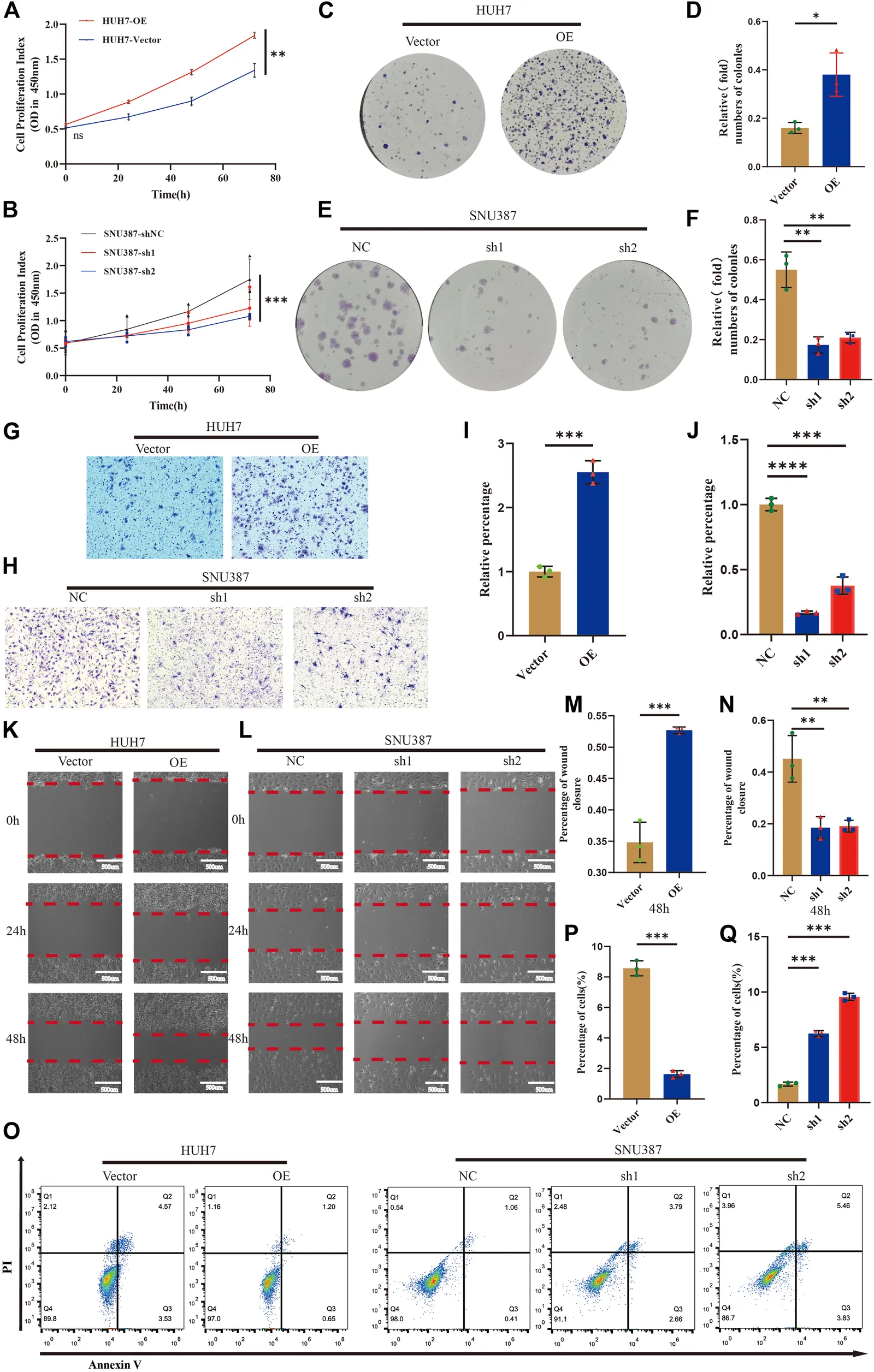
**ITGB5 enhances proliferation, clonogenicity, invasion and migration while suppressing apoptosis in HCC cells.***A* and *B*, CCK-8 cell viability assays detecting proliferative capacity of ITGB5-overexpressing HUH7 cells (*A*) and ITGB5-knockdown SNU387 cells (*B*); *C* and *D*, representative images (*C*) and quantitative analysis (*D*) of colony formation assays in HUH7 cells; *E* and *F*, representative images (*E*) and quantitative analysis (*F*) of colony formation assays in SNU387 cells; *G* and *J*, transwell invasion assays: the number of invaded HUH7 cells (*G* and *I*) and SNU387 cells (*H* and *J*); *K and N*, wound-healing migration assays: the migration rate of HUH7 cells (*K* and *M*) and SNU387 cells (*L* and *N*); *O* and *Q*, apoptosis analysis by Annexin V/PI double-staining flow cytometry: the total apoptotic rate of HUH7 cells (*O* and *P*) and SNU387 cells (*O* and *Q*). Data are expressed as mean ± SD, ∗*p* < 0.05, ∗∗*p* < 0.01, ∗∗∗*p* < 0.001, ∗∗∗∗*p* < 0.001. HCC, hepatocellular carcinoma; ITGB5, identified integrin β5.

**Figure 4:**
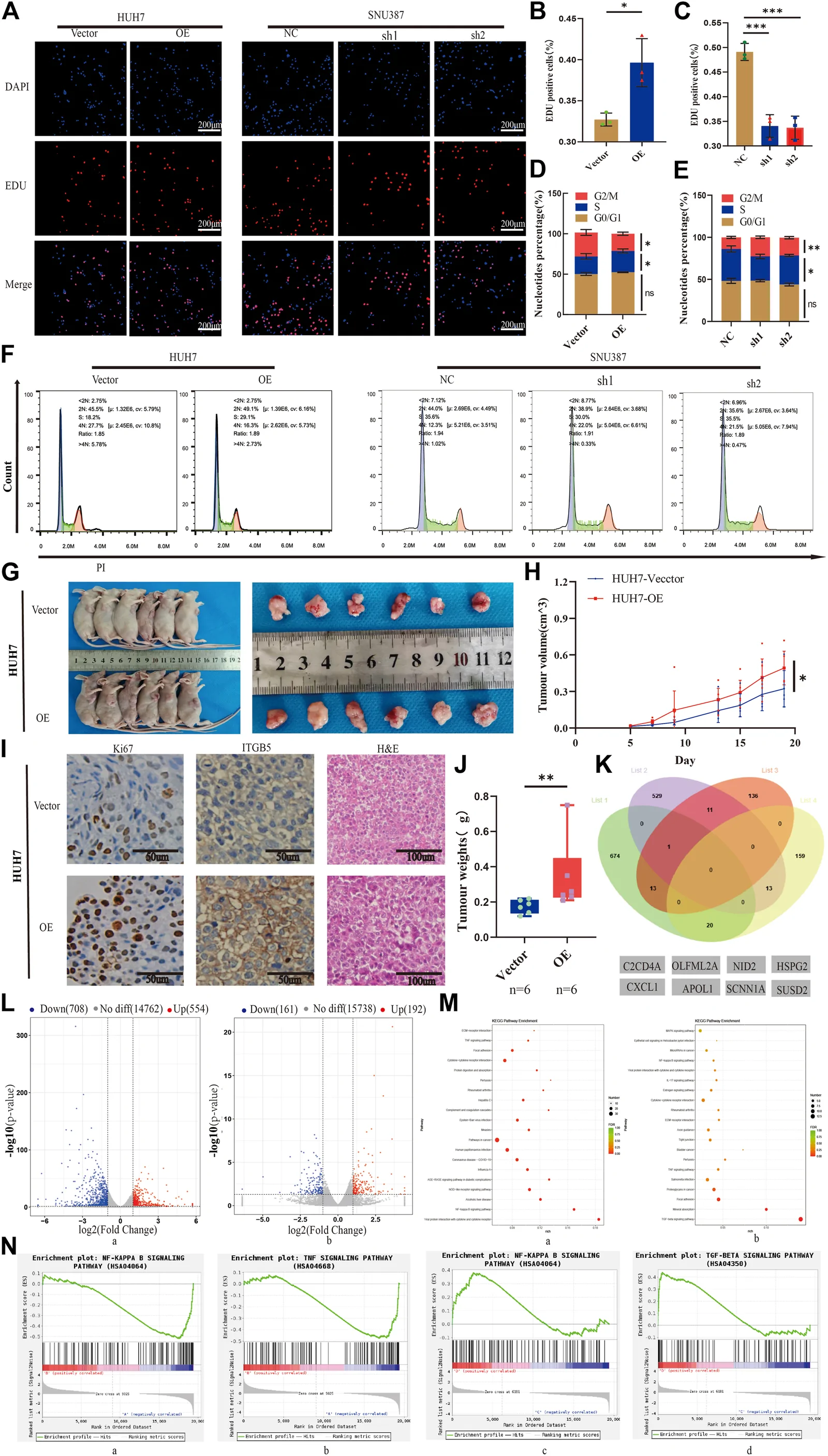
**ITGB5 drives cell-cycle progression and HCC tumor growth *in vivo* and is linked to ECM remodeling, cytokine signaling and inflammatory oncogenic pathways.***A and C*, high-content analysis of EdU/DAPI staining: the EdU-positive proliferation rate in HUH7 cells (*A* and *B*) and SNU387 cells (*A* and *C*); *D* and *F*, cell cycle distribution analyzed by flow cytometry: HUH7 cells (*D* and *F*) and SNU387 cells (*E* and *F*); (*G and H*, mouse model of subcutaneous tumorigenesis using HUH7 cells and corresponding tumor growth curves(n = 6); *I*, immunohistochemical staining (Ki-67, ITGB5) and H&E staining of tumor tissues; (*J*) Statistical analysis of subcutaneous tumor weight; *K*, venn diagram analysis of overlapping DEGs between the ITGB5 overexpression and knockdown groups; *L*, volcano plots of transcriptome sequencing: A total of 1262 DEGs (708 downregulated, 554 upregulated) were identified in the ITGB5 knockdown group (*a*), and 353 DEGs (192 upregulated, 161 downregulated) were identified in the overexpression group (*b*); *M*, KEGG pathway enrichment analysis: DEGs in the ITGB5 knockdown group (*a*) were enriched in signaling pathways including ECM-receptor interaction. Those in the overexpression group (*b*) were enriched in tumor-promoting pathways such as the cytokine-cytokine receptor interaction, ECM-receptor interaction, and TNF signaling pathway; *N*, GSEA enrichment analysis: DEGs in the ITGB5 knockdown (*a* and *b*) and overexpression (*c* and *d*) groups were significantly enriched in NF-κB, TNF and other signaling pathways. Data are expressed as mean ± SD, ∗*p* < 0.05, ∗∗*p* < 0.01, ∗∗∗*p* < 0.001. DEG, differentially expressed gene; GSEA, gene set enrichment analysis; HCC, hepatocellular carcinoma; ITGB5, identified integrin β5.

Gain- and loss-of-function assays demonstrated a tumor-promoting role of ITGB5 in HCC. ITGB5 overexpression significantly accelerated HUH7 cell proliferation(Fig. 3*A*), increased EdU incorporation(Fig. 4, *A* and *B*), enhanced colony formation(Fig. 3, *C* and *D*), promoted invasion(Fig. 3, *G* and *I*) and migration(Fig. 3, *K* and *M*), and reduced apoptosis(Fig. 3, *O* and *P*). Conversely, ITGB5 knockdown in SNU387 cells markedly impaired proliferation(Fig. 3*B*), EdU incorporation (Fig. 4, *A* and *C*), clonogenicity (Fig. 3, *E* and *F*), invasion(Fig. 3, *H* and *J*) and migration (Fig. 3, *L* and *N*) while increasing apoptotic cell death(Fig. 3, *O* and *Q*).

Cell-cycle analysis further showed that ITGB5 overexpression increased the proportion of cells in S phase and reduced the G2/M fraction, indicating enhanced cell-cycle progression(Fig. 4, *D* and *F*). In contrast, ITGB5 knockdown decreased S-phase cells and increased the G2/M population, consistent with cell-cycle arrest (Fig. 4, *E* and *F*). These results establish ITGB5 as an oncogenic regulator of HCC cell growth and metastatic potential.

*In vivo*, subcutaneous xenografts derived from ITGB5-overexpressing HUH7 cells grew significantly faster (Fig. 4, *G* and *H*) and formed larger tumors(Fig. 4*J*) than control cells. Tumors in the ITGB5-overexpression group showed increased Ki-67 staining and more pronounced cellular atypia by H&E staining (Fig. 4*I*), confirming that ITGB5 enhances HCC tumorigenicity *in vivo*.

### Transcriptomic profiling reveals ITGB5-associated oncogenic programs

RNA sequencing following ITGB5 manipulation revealed broad transcriptional reprogramming. ITGB5 knockdown resulted in 1262 DEGs (Fig. 4*L*(*a*)), including 708 downregulated and 554 upregulated genes, whereas ITGB5 overexpression induced 353 DEGs (Fig. 4*L*(*b*)), including 192 upregulated and 161 downregulated genes. Venn diagram analysis (Fig. 4*K*) showed key candidate downstream genes included C2CD4A, CXCL1, HSPG2 and NID2, suggesting that ITGB5 regulates both tumor-intrinsic and microenvironment-related programs.

KEGG enrichment showed that DEGs in the ITGB5-knockdown group were enriched in ECM-receptor interaction, cytokine-cytokine receptor interaction, TNF signaling, NF-κB signaling and pathways in cancer (Fig. 4*M* (*a*)). DEGs induced by ITGB5 overexpression were enriched in MAPK signaling, ECM-receptor interaction, cytokine signaling, TNF/NF-κB signaling, proteoglycans in cancer and TGF-β signaling (Fig. 4*M*(*b*)). GSEA consistently highlighted NF-κB and TNF pathways in both knockdown ((Fig. 4*N* (*a*) and Fig. 4*N* (*b*))) and overexpression((Fig. 4*N* (*c*) and Fig. 4*N* (*d*))) settings. These findings support a model in which ITGB5 drives HCC progression through coordinated regulation of ECM remodeling, inflammatory signaling and oncogenic pathway activation.

### Anti-ITGB5-Nb#2 specifically binds ITGB5 with high affinity

**Figure 5:**
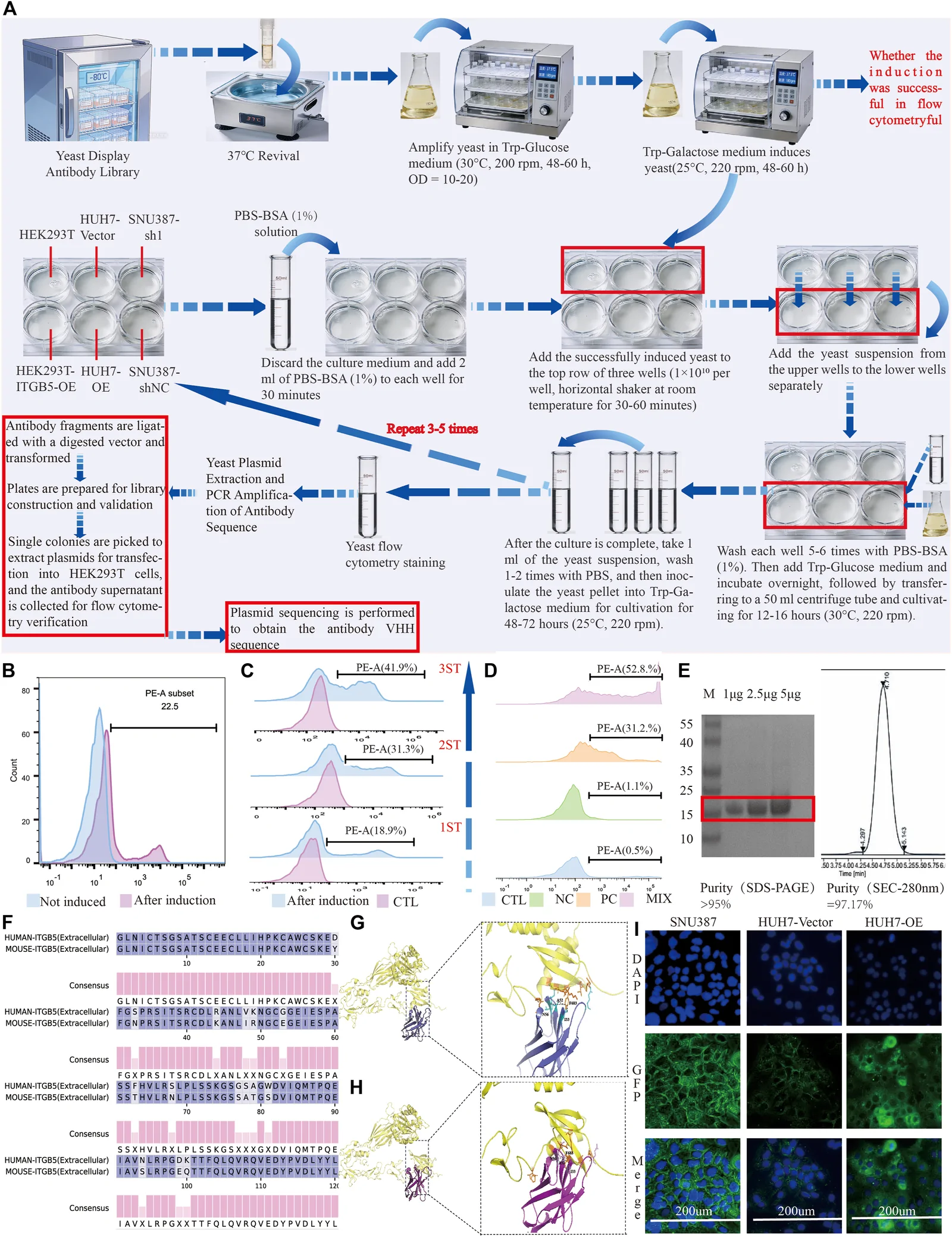
**Screening, biochemical characterization and binding validation of the ITGB5-targeting nanobody Anti-ITGB5 (Nb)#2.***A*, schematic diagram illustrating the overall workflow for screening ITGB5-targeted nanobodies based on yeast surface display technology; *B*, flow cytometry analysis to confirm successful induction of nanobody expression on yeast cells; *C*, flow cytometry quantification of positive yeast clone proportions after three successive rounds of biopanning; *D*, flow cytometric comparison of antigen-binding performance between the enriched nanobody library and commercial anti-ITGB5 antibody; *E*, purity identification of Anti-ITGB5 (Nb)#2 nanobody: SDS-PAGE (*left*) and SEC-HPLC (*right*); *F*, sequence alignment of the extracellular domain amino acid sequences of human and mouse ITGB5 protein; *G* and *H*, molecular docking simulation between ITGB5 protein and Anti-ITGB5 (Nb)#2 nanobody. Representative optimal binding conformations of the nanobody bound to human ITGB5 (*G*) and mouse ITGB5 (*H*) are displayed; *I*, immunofluorescence staining assay to confirm the specific binding capacity of the purified nanobody to intact live HCC cells. ITGB5, identified integrin β5.

To develop an ITGB5-targeting biologic, we screened a yeast-display (Fig. 5*A*) nanobody library using sequential negative and positive cell-based panning. After recovery, induction culture was performed in Trp-Galactose medium for 60 h, and flow cytometry showed a positive rate of 22.5% (Fig. 5*B*). Following three rounds of cell panning, the flow cytometry positive rates reached 18.9%, 31.3%, and 41.9%, respectively (Fig. 5*C*). Antibodies expressed from the enriched pooled library bound ITGB5-overexpressing HEK293T cells more efficiently than a commercial antibody (52.8% *versus* 31.2%) (Fig. 5*D*). Indicating successful enrichment of high-affinity binders.

Thirty-two individual clones were evaluated, among which six showed strong binding signals (Fig. S2, *C* and *F*). Functional screening identified AntiITGB5-Nb#2 as the only clone with pronounced inhibitory activity against ITGB5-high SNU387 cells (Fig. S1*G*). The purified nanobody displayed >95% purity by SDS-PAGE and SEC-HPLC(Fig. 5*E*).

Sequence alignment demonstrated high conservation between human and murine ITGB5 extracellular domains, supporting cross-species applicability(partial alignment shown; Fig. 5*F*). Molecular docking predicted stable interaction between Anti-ITGB5-Nb#2 and both human and mouse ITGB5. PLIP analysis identified several shared predicted interaction pairs in both human and murine ITGB5-VHH models. On the ITGB5 side, the human and murine complexes share eight predicted interface residues, namely P629, T630, C635, K674, L681, F683, V691 and E713. The consistency of these shared residues across human and murine ITGB5 indicates partial conservation of this predicted binding region. On the VHH side, five interacting residues are common to both models: I33, A52, N56, T57 and Q104. Representative interactions include a predicted hydrogen bond between F683 and VHH-N56, as well as predicted hydrophobic contacts between F683 and VHH-I33/A52. This demonstrates that residue F683 resides within the conserved interface region of both human and murine ITGB5 and forms contacts with the VHH in both docking models, marking it as a candidate core interface residue (Fig. 5, *G* and *H*). Under physiological conditions, ITGB5 functions as a αvβ5 heterodimer. Therefore, molecular docking based on isolated monomeric ITGB5 only provides preliminary structural predictions, which require further experimental verification in future studies.

**Figure 6:**
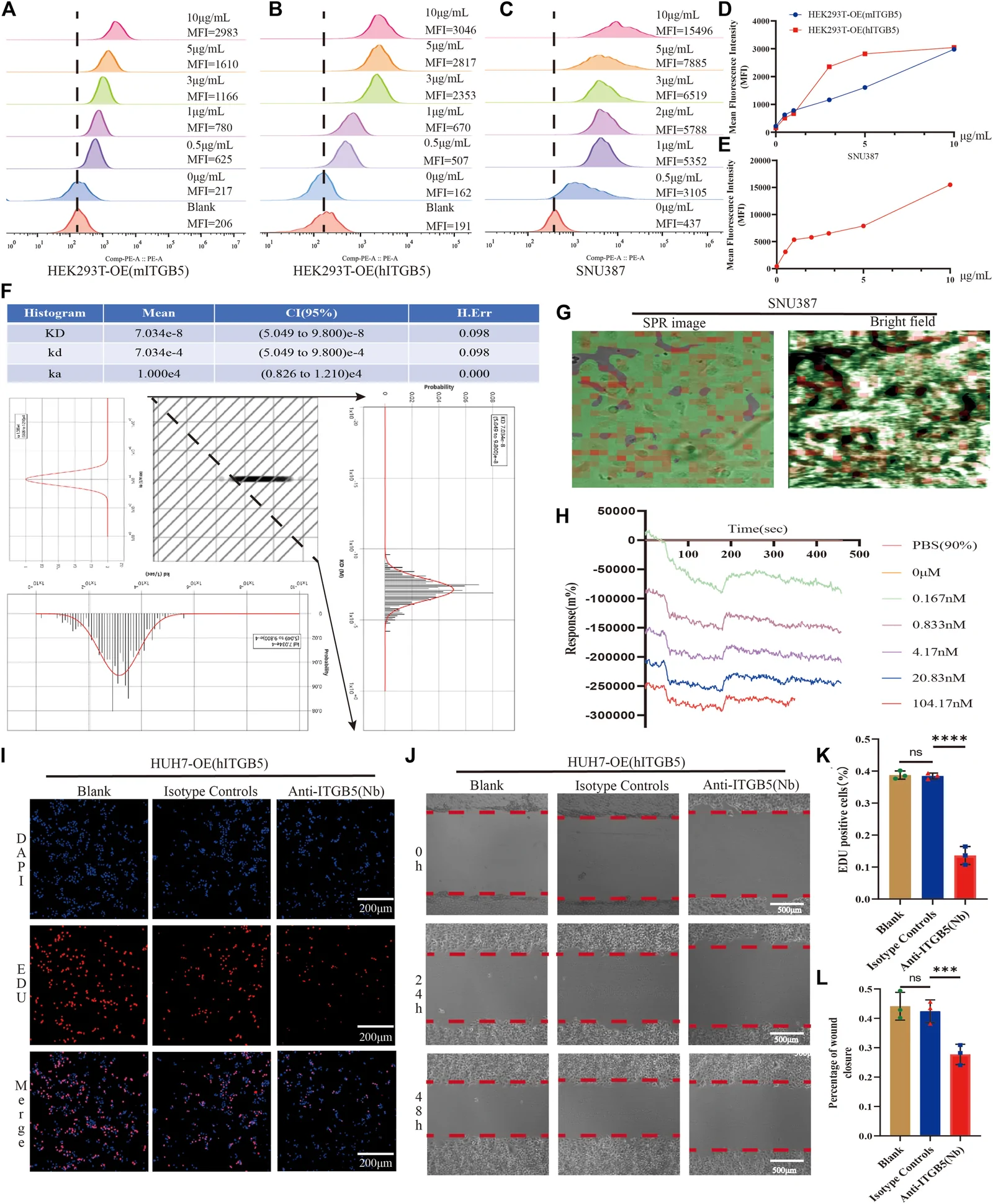
**Anti-ITGB5 (Nb)#2 exhibits high-affinity binding to human and mouse ITGB5 and suppresses malignant phenotypes of HCC cells *in vitro***. *A* and *C*, flow cytometry-based binding assays showing dose-dependent binding of Anti-ITGB5 (Nb)#2 to three cell types: mITGB5-overexpressing HEK293T cells (*A*), hITGB5-overexpressing HEK293T cells (*B*), and SNU387 HCC cells with high endogenous ITGB5 expression (*C*); *D* and *E*, quantitative statistics of mean MFI corresponding to the flow cytometric binding curves shown in panels (*A* and *C*, *F*, SPR sensorgram for kinetic affinity measurement between purified Nb#2 and SNU387 cells;(*G*)SPR and bright-field imaging confirming specific binding of the antibody to antigens on the surface of SNU387 cells; *H*, SPR dose-response curve for affinity fitting of Nb#2 against ITGB5 on the surface of SNU387 cells; *I and K*, EdU cell proliferation assay detecting the proportion of EdU-positive proliferative cells in hITGB5-overexpressing HUH7 (HUH7-OE) cells after Nb#2 treatment; *J and L*, wound-healing migration assay measuring scratch closure rate to assess horizontal migratory capacity of HUH7-OE cells treated with Anti-ITGB5 (Nb)#2. Data are presented as mean ± SD from independent biological replicates. Statistical significance was determined using unpaired two-tailed Student’s *t* test, one-way ANOVA or two-way ANOVA, as appropriate. Dose-dependent binding curves and SPR response analyses were fitted using standard nonlinear regression models where applicable. *p* < 0.05 was considered statistically significant., ∗*p* < 0.05, ∗∗*p* < 0.01, ∗∗∗*p* < 0.001, ∗∗∗∗*p* < 0.0001, not significant (*p* > 0.05). HCC, hepatocellular carcinoma; ITGB5, identified integrin β5.

Immunofluorescence showed strong membrane-localized nanobody binding in SNU387 cells and ITGB5-overexpressing HUH7 cells, but weak staining in ITGB5-low HUH7-vector controls (Fig. 5*I*). Flow cytometry confirmed dose-dependent binding of Anti-ITGB5-Nb#2 to HEK293T cells overexpressing human(hITGB5, Fig. 6*B*) or murine ITGB5(mITGB5, Fig. 6*A*) and to endogenous ITGB5-high SNU387 cells (Fig. 6*C*). Notably, the MFI elevation was most prominent in SNU387 cells, reaching 15,496 at 10 μg/ml antibody, far exceeding that of the blank control (MFI=437) (Fig. 6, *D* and *E*). Surface plasmon resonance (SPR) further demonstrated high-affinity binding, with a KD of 7.034 × 10^−8^ M, an association rate constant (ka) of 1.000 × 10^4^ M^−1^ s^−1^ and a dissociation rate constant (kd) of 7.034 × 10^−4^ s^−1^(Fig. 6, *F*–*H*). Together, these data establish Anti-ITGB5-Nb#2 as a specific and high-affinity ITGB5-targeting nanobody.

### Anti-ITGB5-Nb#2 suppresses HCC cell growth and migration *in vitro*

**Figure 7:**
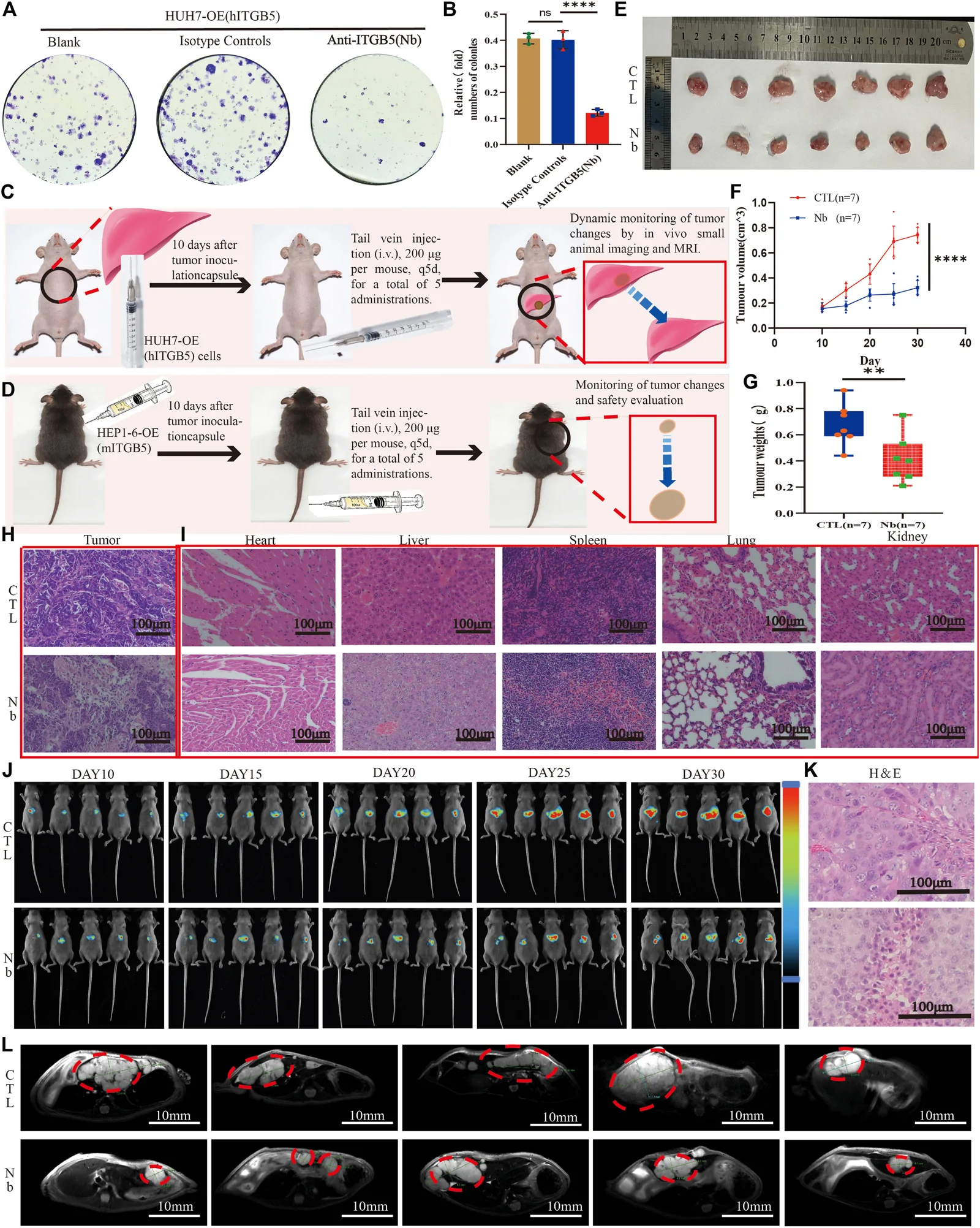
**Anti-ITGB5 (Nb)#2 exerts potent antitumor activity *in vitro* and *in vivo* with a favorable preliminary safety profile.***A* and *B*, representative colony images (*A*) and corresponding quantitative colony counts (*B*) of hITGB5-overexpressing HUH7 cells treated with Anti-ITGB5 (Nb)#2; *C*, schematic diagram of the experimental procedure for the orthotopic HCC model in nude mice; *D*, schematic diagram of the experimental procedure for the subcutaneous HCC model in immunocompetent mice; *E* and *G*, results of subcutaneous tumorigenesis in immunocompetent mice: *E*, gross specimens of tumors from both groups. *F*, tumor growth curves. *G*, statistical quantification of tumor weight measured at the end of the experiment (n = 7 mice per group); *H*, H&E staining of subcutaneous tumor tissues to observe intratumoral histological changes; *I*, H&E histological staining of major visceral organs including heart, liver, spleen, lung and kidney for systemic safety evaluation; *J*, real-time monitoring of orthotopic liver tumor progression *via* small-animal *in vivo* bioluminescence imaging; *K*, H&E staining of orthotopic liver tumor tissues; *L*, dynamic monitoring of orthotopic liver tumor volume by MRI. All quantitative data are presented as mean ± SD. ∗∗*p* < 0.01, ∗∗∗∗*p* < 0.0001, ns, not significant (*p* > 0.05). HCC, hepatocellular carcinoma; ITGB5, identified integrin β5.

**Fig. S3:**
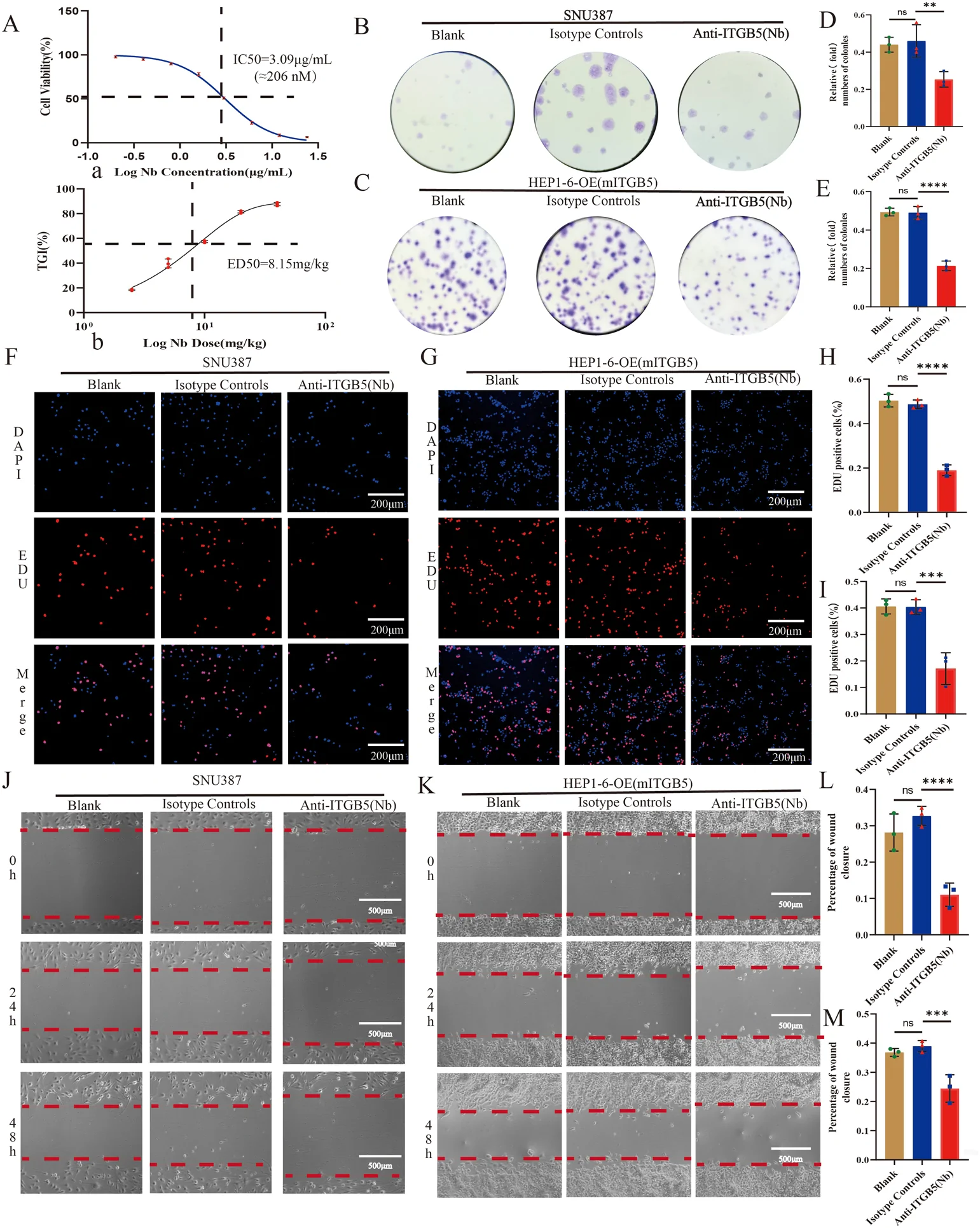


Anti-ITGB5-Nb#2 dose-dependently inhibited proliferation of SNU387 cells, with an IC_50_ of 3.09 μg/ml, corresponding to approximately 206 nM (Fig. S3*A* (*a*)). The *in vivo* ED50 was calculated as 8.15 mg/kg (Fig. S3*A* (*b*)). Treatment with 10 μg/ml Anti-ITGB5-Nb#2 significantly reduced EdU incorporation in ITGB5-overexpressing HUH7 cells (Fig. 6, *I* and *K*),SNU387 cells (Fig. S3, *F* and *H*) and murine ITGB5-overexpressing HEP1-6 cells (Fig. S3, *G* and *I*). Wound-healing assays showed that nanobody treatment substantially impaired migration in ITGB5-overexpressing HUH7 cells(Fig. 6, *J* and *L*), SNU387 cells (Fig. S3, *J* and *L*) and murine ITGB5-overexpressing HEP1-6 cells (Fig. S3, *K* and *M*). Colony formation assays further demonstrated reduced clonogenic growth after Anti-ITGB5-Nb#2 treatment, ITGB5-overexpressing HUH7 cells (Fig. 7, *A* and *B*), SNU387 cells (Fig. s3B and s3D) and murine ITGB5-overexpressing HEP1-6 cells (Fig. S3, *C* and *E*). These results indicate that ITGB5 blockade by Anti-ITGB5-Nb#2 directly suppresses proliferative and migratory capacities of HCC cells.

### Anti-ITGB5-Nb#2 inhibits HCC growth *in vivo*

The therapeutic efficacy of Anti-ITGB5-Nb#2 was first evaluated in an orthotopic human HCC model generated by intrahepatic implantation of HUH7-OE cells (Fig. 7*C*) in nude mice. Starting 10 days after implantation, mice received intravenous Anti-ITGB5-Nb#2 at 200 μg per mouse every 5 days for five doses. *In vivo* imaging (Fig. 7*J*) and magnetic resonance imaging (Fig. 7*L*) demonstrated significantly reduced tumor growth in the treatment group compared with controls. Histological analysis revealed extensive tumor necrosis and decreased tumor cell density after nanobody treatment (Fig. 7*K*), indicating potent antitumor activity against orthotopic HCC.

To assess immune-dependent effects, Anti-ITGB5-Nb#2 was further tested in an immunocompetent C57BL/6 subcutaneous HCC model established with HEP1-6 cells expressing murine ITGB5(Fig. 7*D*). Nanobody treatment markedly suppressed tumor growth compared with control treatment (Fig. 7*E*). At the experimental endpoint, the tumor volume in the control group reached (0.75 ± 0.08) cm^3^, whereas that in the nanobody group was only (0.32 ± 0.05) cm^3^, representing an inhibition rate exceeding 57% (*p* < 0.0001) (Fig. 7*F*). Tumor weight was also significantly lower in the treatment group (mean tumor weight ≈ 0.41 g) than in the control group (≈0.68 g) (*p* < 0.01) (Fig. 7*G*).Histopathological analysis by H&E staining showed dense tumor cell arrangement, marked atypia, and minimal necrosis in control HCC tissues. In contrast, the treatment group exhibited extensive necrotic regions, markedly reduced tumor cell density, and typical necrotic changes including karyopyknosis, karyorrhexis, and cytoplasmic lysis (Fig. 7*H*). These findings histopathologically confirm that Anti-ITGB5 (Nb)#2 effectively induces HCC cell necrosis and inhibits tumor growth *in vivo*, further validating its antitumor activity against murine HCC cells in immunocompetent hosts and providing experimental evidence for its *in vivo* safety and efficacy toward clinical translation.

### Anti-ITGB5-Nb#2 safety evaluation

To evaluate the *in vivo* safety of Anti-ITGB5 (Nb)#2, H&E staining and pathological analysis were performed on major organs including the heart, liver, spleen, lung, and kidney at the experimental endpoint. As shown in Figure 7*I*, the treatment group exhibited intact tissue structure in all organs, with no obvious pathological damage such as inflammatory infiltration, cellular necrosis, or tissue degeneration.

Meanwhile, systematic serum biochemical parameters were measured in mice from the control group and the Anti-ITGB5 (Nb) #2 group at the endpoint of administration (Table 1). The results showed that, compared with the CTL group, no significant differences were observed in core biochemical indices in the treatment group (*p* > 0.05), including ALT, AST, ALP, GGT and bilirubin for liver function, UREA and UA for renal function, TG and CHOL for lipid metabolism, as well as CK and LDH for cardiac function. All parameters remained within normal physiological ranges.

**Table 1:** Effects of Anti-ITGB5 (Nb)#2 on serum biochemical parameters in mice

Test item	Unit	Control group (x¯ ± s)	Treatment group (x¯ ± s)
ALT	U/L	5.3 ± 0.4	6.3 ± 0.3
AST	U/L	14.0 ± 1.6	16.0 ± 2.6
TBil	μmol/L	1.2 ± 0.3	1.1 ± 0.1
DBil	μmol/L	0.3 ± 0.1	0.4 ± 0.0
IBIL	μmol/L	0.6 ± 0.2	0.7 ± 0.2
TP	g/L	4.1 ± 0.3	4.4 ± 0.3
ALB	g/L	0.4 ± 0.1	0.4 ± 0.2
GLB	g/L	3.8 ± 0.3	4.0 ± 0.3
A/G	-	0.10 ± 0.01	0.10 ± 0.01
ALP	U/L	64.3 ± 4.2	57.7 ± 1.2
γ- GGT	U/L	0.0 ± 0.0	0.0 ± 0.0
CHE	U/L	531.0 ± 9.6	565.0 ± 8.7
UREA	mmol/L	0.88 ± 0.02	0.94 ± 0.07
UA	μmol/L	29.0 ± 2.3	15.0 ± 1.8
GLU	mmol/L	0.64 ± 0.18	0.55 ± 0.18
TG	mmol/L	0.14 ± 0.05	0.12 ± 0.06
CHOL	mmol/L	0.09 ± 0.05	0.07 ± 0.01
LDL-C	mmol/L	0.04 ± 0.03	0.03 ± 0.01
CK	U/L	67.3 ± 4.6	74.7 ± 5.6
LDH	U/L	175.3 ± 8.0	162.7 ± 7.5
HBDH	U/L	37.0 ± 3.5	39.0 ± 4.4
Ca	mmol/L	0.25 ± 0.01	0.19 ± 0.03
Mg	mmol/L	0.17 ± 0.02	0.17 ± 0.04
P	mmol/L	0.22 ± 0.03	0.18 ± 0.03
Fe	μmol/L	3.7 ± 1.4	3.5 ± 1.9

Note: Data are presented as mean ± standard deviation of results from three mice. No statistically significant difference was observed between the two groups (*p* > 0.05).

In summary, the Anti-ITGB5 (Nb)#2 developed in this study achieves significant antitumor efficacy *in vivo* without inducing systemic biochemical abnormalities in mice, showing excellent *in vivo* safety and providing solid safety data for its future clinical application.

### Anti-ITGB5-Nb#2 remodels antitumor immunity *in vivo*

**Fig. S4:**
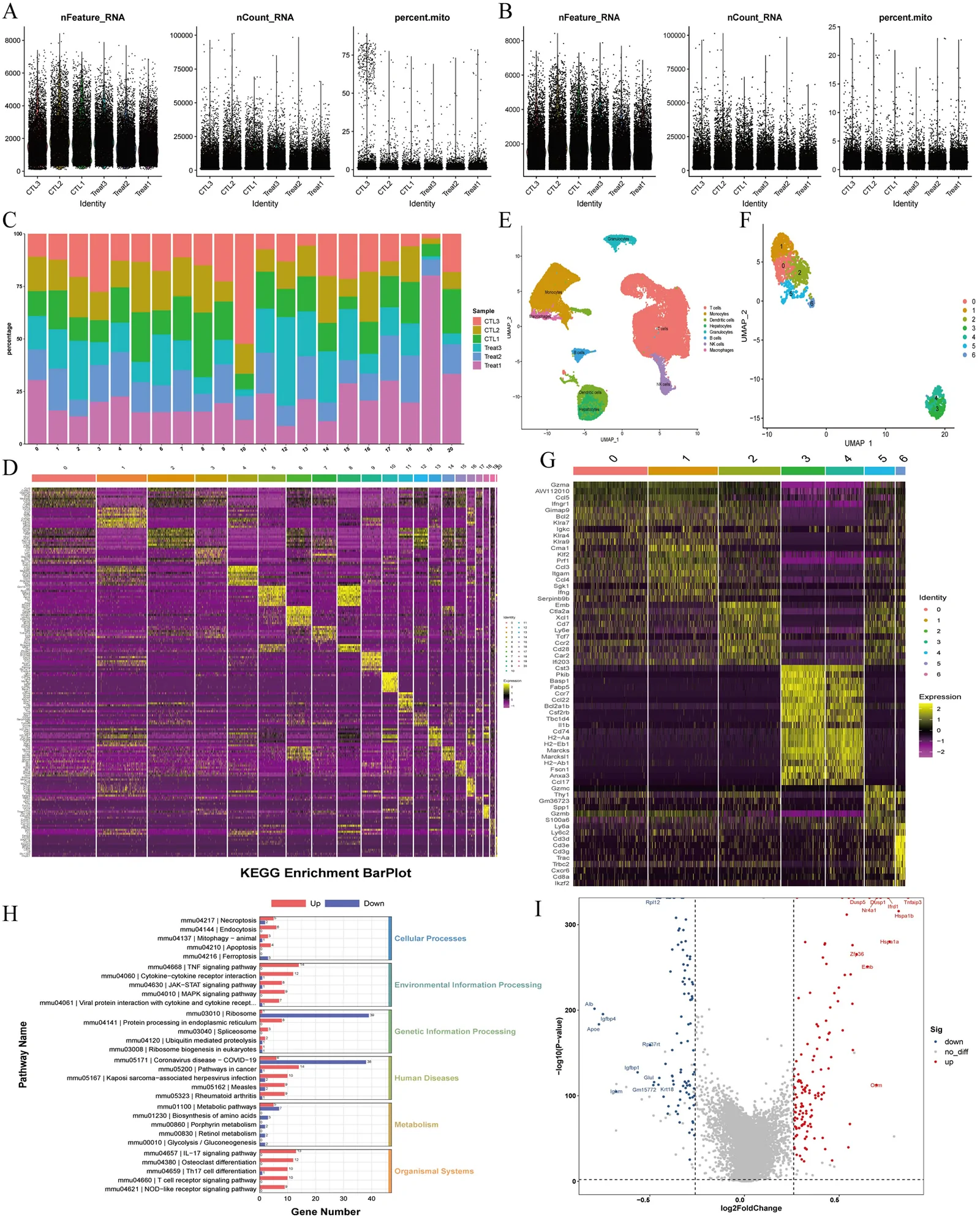


To define the immune mechanisms underlying the *in vivo* efficacy of Anti-ITGB5-Nb#2, we performed scRNA-seq on tumors from immunocompetent mice treated with Anti-ITGB5-Nb#2 or control antibody, with three biological replicates per group. After stringent quality control, 19,573 cells from control tumors and 21,144 cells from treated tumors were retained for downstream analysis. All samples showed high sequencing quality, with concentrated distributions of detected genes and unique molecular identifier (UMI) counts and mitochondrial gene proportions below 5% (Fig. S4, *A* and *B*).

**Figure 8:**
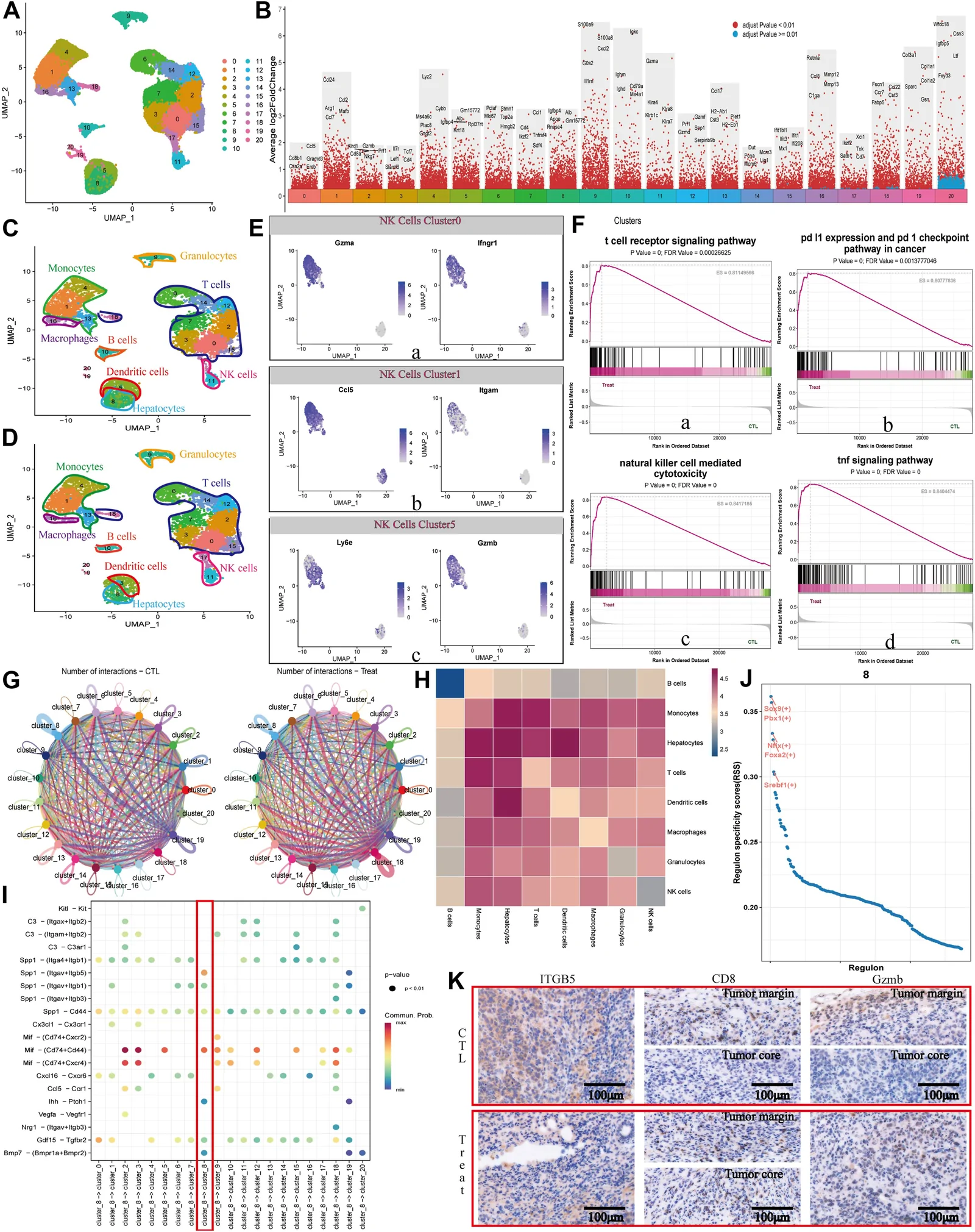
**scRNA-seq reveals that Anti-ITGB5-Nb#2 remodels antitumor immunity *in vivo*.***A*, UMAP dimensional reduction plots displaying single-cell transcriptional clusters derived from subcutaneous tumor tissues of vehicle control and Anti-ITGB5-Nb#2-treated C57 mice; *B*, heatmap illustrating the top five highly expressed marker genes for each distinct cell cluster identified in the dataset; *C* and *D*, SingleR-based cell-type annotation and proportional analysis of major cell populations in control (*C*) and treated tumors (*D*); *E*, NK-cell subclustering showing functionally distinct subsets, including cytotoxic populations expressing *Gzma*, *Ifngr1*, *Ly6e* and *Gzmb*, and an activated/regulatory subset expressing *Ccl5* and *Itgam*; *F*, GSEA of DEGs after Anti-ITGB5-Nb#2 treatment; *G*, cell–cell communication network showing treatment-induced remodeling of intercellular signaling; *H* heatmap quantifying communication strength among major cell types, including hepatocytes, T cells, macrophages, dendritic cells and NK cells; *I*, bubble plot summarizing significantly changed ligand-receptor interaction pairs secreted by Cluster eight malignant tumor cells as signal sender; *J*, pySCENIC regulon analysis comparing differential transcription factor regulatory activity within tumor cells between control and treatment groups; *K*, immunohistochemical validation of ITGB5, CD8 and Gzmb expression in control and treated tumors. DEG, differentially expressed gene; GSEA, gene set enrichment analysis; ITGB5, identified integrin β5.

Unsupervised clustering identified 21 transcriptionally distinct cell clusters, visualized by UMAP (Fig. 8*A*). The top five highly expressed genes specific to each cluster were identified (Fig. 8*B*). Cluster-specific marker analysis revealed a malignant hepatocellular carcinoma population, Cluster 8, and multiple immune-cell populations, including NK-cell-enriched Clusters 11 and 17. A heatmap of the top 10 DEGs was also generated (Fig. S4*D*), providing a basis for subsequent cell type identification.

Compared with control tumors, Anti-ITGB5-Nb#2-treated tumors showed a marked reduction in Cluster eight and increased proportions of immune-associated clusters, including Clusters 11, 15 and 17 (Fig. S4*C*). SingleR-based annotation further confirmed that tumor tissues mainly contained various cell types, including T cells, NK cells, macrophages, dendritic cells, B cells, and HCC cells (Fig. S4*E*). Treated tumors contained fewer HCC cells and higher fractions of NK cells and selected T-cell subsets than control tumors (Fig. 8, *C* and *D*).These data indicate that Anti-ITGB5-Nb#2 reduces the malignant tumor-cell compartment while promoting immune-cell infiltration within the HCC microenvironment.

Given the expansion of NK cells after treatment, we further subclustered the annotated NK-cell population. The results showed that NK cells could be further divided into several functionally distinct subsets (Fig. S4*F*). Heatmap analysis of DEGs across subsets (Fig. S4*G*) revealed remarkable functional heterogeneity among different NK cell subsets. Cluster 0 highly expressed cytotoxicity-associated genes, including Gzma and Ifngr1 (Fig. 8*E*(*a*)). Cluster one was enriched for Ccl5 and Itgam, suggesting an activated or regulatory phenotype (Fig. 8*E*(*b*)). Cluster five expressed Ly6e and Gzmb, consistent with cytotoxic effector activity (Fig. 8*E*(*c*)). Other subsets each highly expressed a series of related genes, such as regulatory genes and chemokine receptor genes. These results clearly demonstrate the gene expression heterogeneity of NK cell subsets, indicating that NK cells in the TME are not a homogeneous population but consist of multiple functional subsets. This also suggests that other immune cells (such as T cells and macrophages) are similarly heterogeneous, with different subsets exerting either pro-tumor or anti-tumor functions in cancer immunotherapy.

We next assessed transcriptional programs altered by Anti-ITGB5-Nb#2 treatment. Differential expression analysis revealed broad transcriptional remodeling in treated tumors compared with controls (Fig. S4*I*). KEGG enrichment analysis showed that DEGs were mainly involved in immune regulation and cell death-related pathways, including cytokine–cytokine receptor interaction, TNF signaling, IL-17 signaling, Th17-cell differentiation, T-cell receptor signaling and apoptosis (Fig. S4*H*). Consistently, GSEA demonstrated significant enrichment of T-cell receptor signaling, PD-L1 expression and PD-1 checkpoint pathway in cancer, natural killer cell-mediated cytotoxicity and TNF signaling in treated tumors (Fig. 8*F*). These pathways were concordant with those identified after ITGB5 perturbation in bulk transcriptomic analyses, supporting a mechanistic link between ITGB5 blockade, immune activation and tumor-cell elimination.

### Pseudotime analysis

To resolve dynamic transcriptional changes induced by Anti-ITGB5-Nb#2, we performed pseudotime trajectory analysis. The top DEGs were organized into distinct expression modules along the pseudotime axis (Fig. S5*A*). One module contained immune checkpoint and immunoregulatory genes, including *Ctla4*, *Cd274/Pd-l1*, *Pdcd1/Pd-1* and *Havcr2/Tim-3*, together with tumor-associated regulators such as *Bcl2* and *Stat4*. These genes progressively decreased along pseudotime, suggesting attenuation of immunosuppressive programs after treatment. Cluster two contained key molecules including *G0s2*, *Cavin2*, and *Serpinb7*, whose expression was significantly upregulated in the middle stage of pseudotime, indicating that antibody treatment effectively activated antitumor responses. In Cluster 3, upregulated expression of *Col3a1*, *Fn1*, and other genes indicated extracellular matrix remodeling and tissue repair. Expression of inflammation-related genes such as *Ptgs2*, *Fcgr2b*, and *Fcgr3* reflected sustained inflammatory responses and immune microenvironment regulation. Expression of *Ier5*, *Creg1*, and other genes indicated stress responses and adaptive survival mechanisms of tumor cells under therapeutic pressure. Genes including *Gpr35* and *Pla2g4a* were involved in cell signal transduction and metabolic reprogramming. The dynamic expression of these molecules not only reflects the impact of treatment on the tumor microenvironment but also provides important molecular evidence for evaluating therapeutic efficacy, predicting prognosis, and optimizing therapeutic strategies.

### CytoTRACE analysis

CytoTRACE analysis further showed that Cluster eight tumor cells exhibited high differentiation-potential scores, consistent with a poorly differentiated, stem-like and highly malignant state, whereas immune populations displayed broader differentiation states (Fig. S5*B*). These findings suggest that Anti-ITGB5-Nb#2 dynamically reshapes immunosuppressive, inflammatory and stromal programs within the tumor microenvironment.

### Cell-cell communication analysis

T We then investigated whether Anti-ITGB5-Nb#2 altered intercellular communication. Global cell–cell interaction strength was increased after treatment compared with control tumors (control, 0.74; treatment, 0.994), indicating extensive remodeling of the communication network (Fig. S5*C*). Differential communication analysis showed enhanced signaling pathways related to immune activation, antigen presentation and chemokine signaling in treated tumors, whereas fewer communication pathways were preferentially active in control tumors (Fig. S5*D*). In control tumors, Cluster eight formed dense communication networks with multiple immune and stromal populations, indicating that tumor cells acted as a central signaling hub within the immunosuppressive microenvironment (Fig. 8*G*). After Anti-ITGB5-Nb#2 treatment, interactions between Cluster eight and other cell subsets were markedly reduced, while immune-cell communication was selectively preserved or strengthened.

### Using cell-cell communication analysis

Quantitative analysis of incoming and outgoing signaling further showed reduced communication capacity of Cluster eight and increased activity of several immune-associated clusters after treatment (Fig. S5, *E* and *F*). These results suggest that Anti-ITGB5-Nb#2 disrupts tumor-centered immunosuppressive signaling while reinforcing immune-effector communication.

### Ligand–receptor analysis

A communication heatmap showed strong interactions among hepatocytes, T cells, macrophages, dendritic cells and NK cells after treatment (Fig. 8*H*). When Cluster eight tumor cells were defined as the signaling sender, Anti-ITGB5-Nb#2 markedly reduced multiple tumor-associated ligand–receptor interactions, including autocrine or paracrine *Itgav–Itgb5* signaling and immunosuppression- or invasion-related pathways such as *Spp1–Cd44* and *Mif*–(*Cd74*+*Cd44*) (Fig. 8*I*). These data provide single-cell ligand–receptor evidence that Anti-ITGB5-Nb#2 blocks aberrant tumor-derived communication networks in the HCC microenvironment.

### Transcription factor analysis

pySCENIC regulon analysis was used to evaluate transcription factor activity after treatment. In control tumors, Cluster eight displayed high activity of transcription factors associated with tumor stemness, proliferation, invasion and ITGB5-related malignant phenotypes, including Foxa2, Sox9 and Pbx1 (Figs. 8*J*, S5*G*). UMAP visualization of transcription factor AUC distribution confirmed that these core transcription factors were specifically and highly activated in Cluster eight tumor cells (Fig. S5, *H* and *J*).Anti-ITGB5-Nb#2 treatment reduced the activity of these pro-tumor regulons in tumor cells, while enhancing immune activation-associated transcriptional programs in immune-cell populations (Fig. 8*J*). These findings indicate that ITGB5 blockade suppresses tumor-intrinsic malignant regulatory circuits and promotes antitumor immune activation.

### Immunohistochemical validation

Immunohistochemistry further validated the scRNA-seq findings at the tissue level. Control tumors showed strong ITGB5 staining in tumor cells, whereas CD8^+^ and Gzmb^+^ cells were sparse and largely restricted to the tumor margin, consistent with an immune-excluded phenotype. In contrast, Anti-ITGB5-Nb#2-treated tumors showed reduced ITGB5 staining and increased infiltration of CD8^+^ and Gzmb^+^ cells in both the tumor margin and tumor core (Fig. 8*K*). Together, these data demonstrate that Anti-ITGB5-Nb#2 converts the HCC microenvironment from a tumor-dominated, immune-excluded state toward an immune-active state characterized by enhanced NK- and T-cell cytotoxicity, disrupted tumor-derived immunosuppressive communication and suppression of malignant transcriptional programs.

## Discussion

HCC remains a major therapeutic challenge because of its profound molecular heterogeneity, intrinsic resistance to systemic therapy, and highly immunosuppressive tumor microenvironment (18). Although immune checkpoint blockade combined with antiangiogenic therapy has reshaped the treatment landscape of advanced HCC, durable responses are still limited to a subset of patients, and clinically actionable biomarkers for patient stratification remain insufficient. Therefore, identifying tumor-intrinsic drivers that are simultaneously targetable and functionally linked to immune remodeling is essential for the development of next-generation HCC immunotherapies (19).

In this study, we identify ITGB5 as a previously underappreciated oncogenic and immunoregulatory driver in HCC. Multi-cohort transcriptomic analyses across TCGA and ICGC cohorts, combined with clinical pathological correlation analysis, consistently verified that *ITGB5* is aberrantly upregulated in HCC tissues and serves as an independent adverse prognostic biomarker correlated with advanced pathological grades and malignant tumor progression. Bioinformatic functional enrichment analyses further revealed that ITGB5-related genes are prominently enriched in extracellular matrix organization and transmembrane signaling functions, and multiple core tumor-promoting cascades, including TNF/NF-κB, MAPK, TGF-β, WNT, VEGF, and Toll-like receptor signaling, are significantly activated in ITGB5-high HCC. These pathway signatures strongly support that ITGB5 acts as a central upstream regulator driving the malignant phenotypic transformation of HCC. Notably, our immune correlation and infiltration analyses further demonstrated that elevated *ITGB5* expression is tightly coupled with increased expression of multiple immune checkpoint molecules and immunosuppressive mediators, as well as robust enrichment of tumor-associated macrophages and significant depletion of cytotoxic CD8^+^ T cells and γδ T cells. This specific immune landscape indicates that ITGB5 facilitates the formation of an immunosuppressive TME, thereby promoting tumor immune evasion in HCC. In addition, drug sensitivity analysis further revealed that ITGB5 overexpression confers broad resistance to first-line HCC targeted drugs and chemotherapeutic agents, including sorafenib and oxaliplatin, while predicting differential responsiveness to trametinib and 5-fluorouracil, highlighting its potential value for clinical drug stratification and personalized therapy. Functionally, ITGB5 promoted HCC cell proliferation, clonogenicity, invasion and migration, inhibited apoptosis, and enhanced tumor growth *in vivo*. Transcriptomic analyses further linked ITGB5 to extracellular matrix remodeling, cytokine–cytokine receptor interaction, TNF/NF-κB, MAPK and TGF-β signaling, supporting its role as a nodal regulator connecting malignant tumor behavior with immune microenvironmental remodeling.

A major translational advance of this work is the generation of a therapeutically functional anti-ITGB5 nanobody, Anti-ITGB5-Nb#2. Unlike conventional monoclonal antibodies, nanobodies possess a small molecular size, favorable tissue penetration, structural stability and engineering flexibility, making them particularly suitable for solid tumors with dense stromal barriers such as HCC. Anti-ITGB5-Nb#2 showed specific binding to both human and murine ITGB5, recognized cell-surface ITGB5 in HCC cells, and exhibited high-affinity binding by flow cytometry, immunofluorescence and SPR analyses. Functionally, this nanobody inhibited proliferation, colony formation and migration of ITGB5-high HCC cells *in vitro* and suppressed tumor growth in both orthotopic and immunocompetent mouse models. These findings provide proof-of-concept evidence that ITGB5 is not merely a prognostic biomarker but a therapeutically exploitable vulnerability in HCC.

Importantly, our single-cell analyses suggest that Anti-ITGB5-Nb#2 exerts antitumor activity through both tumor-intrinsic and immune-mediated mechanisms. Treatment reduced the malignant HCC cell compartment while increasing NK-cell and T-cell populations within the tumor microenvironment. NK-cell subclustering revealed enrichment of cytotoxic programs marked by Gzma, Gzmb, Ifngr1 and related effector genes. Pathway enrichment analyses further showed activation of T-cell receptor signaling, natural killer cell-mediated cytotoxicity, TNF signaling, apoptosis and immune checkpoint-related pathways. Pseudotime and cell–cell communication analyses indicated that Anti-ITGB5-Nb#2 attenuated immunosuppressive checkpoint programs, disrupted tumor-centered ligand–receptor networks including Itgav–Itgb5, Spp1–Cd44 and Mif–Cd74/Cd44, and shifted the tumor microenvironment from a tumor-dominated, immune-excluded state toward an immune-active state. These observations were supported by immunohistochemical evidence showing reduced ITGB5 expression and increased CD4^+^ and Gzma^+^ cell infiltration in treated tumors. Collectively, these data position ITGB5 blockade as a strategy capable of simultaneously suppressing malignant signaling and reinvigorating antitumor immunity.

From a translational perspective, ITGB5-targeted nanobody therapy may offer several potential clinical advantages. First, ITGB5 is a membrane-localized protein with limited expression in normal liver tissues but elevated expression in HCC, supporting its accessibility and therapeutic selectivity. Second, its association with drug resistance suggests that ITGB5 blockade may be valuable for patients with resistance to tyrosine kinase inhibitors, anti-angiogenic therapy or ICIs. Third, because ITGB5 is linked to extracellular matrix remodeling, macrophage infiltration and cytotoxic lymphocyte exclusion, Anti-ITGB5-Nb#2 may synergize with PD-1/PD-L1 inhibitors, VEGF/VEGFR blockade, TKIs or macrophage-targeting strategies. Therefore, Anti-ITGB5-Nb#2 could potentially serve not only as a monotherapy for ITGB5-high HCC but also as a component of rational combination regimens designed to overcome immune exclusion and therapeutic resistance.

Nevertheless, several limitations should be acknowledged before clinical translation. First, although Anti-ITGB5-Nb#2 demonstrated antitumor activity in mouse models, the pharmacokinetic and pharmacodynamic properties of this nanobody remain incompletely defined. Because unmodified nanobodies are rapidly cleared by renal filtration due to their small size, there *in vivo* half-life, tumor retention, biodistribution and optimal dosing schedule require systematic characterization. Half-life extension strategies, such as albumin-binding domain fusion, Fc fusion, PEGylation or multivalent formatting, may be needed to improve therapeutic exposure while preserving tumor penetration (20, 21, 22, 23). Second, the safety profile of ITGB5 blockade requires deeper evaluation. ITGB5 participates in integrin-mediated cell adhesion, extracellular matrix interaction, angiogenesis, tissue remodeling and immune regulation. Although preliminary histological analyses did not suggest overt toxicity, comprehensive toxicology studies in multiple organs, including liver, kidney, lung, vascular endothelium and immune organs, are essential. In particular, potential effects on wound healing, liver regeneration, platelet function, vascular integrity and inflammatory responses should be carefully assessed, as these processes may involve integrin signaling. Third, the current study establishes strong preclinical evidence but does not yet define the precise structural and functional epitope responsible for ITGB5 blockade. Molecular docking predicted potential interaction interfaces, but high-resolution structural validation by cryo-electron microscopy, X-ray crystallography or mutational epitope mapping is required. Such studies would clarify whether Anti-ITGB5-Nb#2 blocks ligand binding, alters integrin conformation, induces receptor internalization or disrupts downstream signaling. This information will be critical for rational affinity maturation, humanization and engineering of next-generation candidates. Fourth, although scRNA-seq revealed immune remodeling after treatment, the causal immune effector populations remain to be functionally validated. Depletion studies targeting NK cells, CD8^+^ T cells, CD4^+^ T cells or macrophages, as well as cytokine-blockade experiments, are needed to determine which immune compartments are indispensable for the therapeutic efficacy of Anti-ITGB5-Nb#2. Spatial transcriptomics, multiplex immunofluorescence and *in situ* ligand–receptor mapping would further clarify how ITGB5 blockade remodels immune-cell localization, tumor–stroma interactions and immune exclusion *in situ*. Fifth, the translational relevance of ITGB5 as a biomarker requires validation in larger and clinically annotated HCC cohorts. Future studies should determine whether ITGB5 expression correlates with response to current first-line regimens such as atezolizumab plus bevacizumab, durvalumab plus tremelimumab, lenvatinib-based combinations or other ICI/TKI strategies (24). Establishing standardized immunohistochemical scoring, defining clinically meaningful cutoffs and integrating ITGB5 with immune signatures, macrophage markers, T-cell exclusion scores and genomic subtypes will be essential for selecting patients most likely to benefit from ITGB5-targeted therapy. Finally, nanobody development offers broad engineering opportunities that remain to be explored. Anti-ITGB5-Nb#2 may be reformatted into bivalent or bispecific nanobodies to increase avidity or recruit immune effector mechanisms. It may also serve as a targeting module for antibody–drug conjugates, radionuclide therapy, CAR-T/NK cell design, nanoparticle delivery or imaging probes for ITGB5-positive lesions (25, 26). Given the membrane localization and tumor enrichment of ITGB5, theranostic development using radiolabeled Anti-ITGB5-Nb#2 may enable non-invasive patient selection, pharmacodynamic monitoring and real-time assessment of target engagement.

In conclusion, this study identifies ITGB5 as a druggable oncogenic and immunoregulatory target in HCC and establishes Anti-ITGB5-Nb#2 as a promising nanobody candidate with direct antitumor activity and immune-remodeling capacity. By disrupting tumor-intrinsic malignant programs and converting the HCC microenvironment toward an immune-active state, ITGB5 blockade provides a rational strategy for improving the efficacy of HCC immunotherapy. Further optimization of nanobody pharmacology, safety, structural mechanism, biomarker-guided patient selection and rational combination regimens will be essential to advance Anti-ITGB5-Nb#2 toward clinical translation.

## Experimental procedures

### Bioinformatic analysis

Pan-cancer expression of ITGB5 was analyzed using TIMER2.0 based on the Cancer Genome Atlas(TCGA) datasets. RNA-sequencing data and clinical information for the TCGA-LIHC cohort were downloaded from the Genomic Data Commons, and external validation was performed using the ICGC database (27). Patients were stratified into ITGB5-high and ITGB5-low groups according to the median expression level. Kaplan-Meier survival analysis and univariate and multivariate Cox regression were performed to evaluate prognostic significance (28).

Protein expression, tissue distribution and subcellular localization of ITGB5 were examined using the Human Protein Atlas. Correlations between ITGB5 and HCC-related molecules, immune checkpoints and immune cell infiltration were assessed using TCGA-LIHC and TIMER2.0 (29). Immune infiltration was estimated using the CIBERSORT algorithm (30). DEGs between ITGB5-high and ITGB5-low tumors were identified using DESeq2, followed by GO, KEGG and GSEA using ClusterProfiler (31).

### Patient specimens and tissue microarray

A total of 140 paired HCC and adjacent non-tumor tissue specimens were used for tissue microarray analysis. Six additional paired fresh HCC and adjacent liver tissues were collected for molecular validation. None of the enrolled patients received preoperative radiotherapy, chemotherapy, hormone therapy or systemic antitumor treatment. The study was approved by the Ethics Committee of the Second Hospital of Lanzhou University (No. 2025A-412), and written informed consent was obtained from all participants. All human subject experiments in this study were performed in accordance with the ethical standards laid down in the Declaration of Helsinki.

### Cell culture and genetic manipulation

HEK293T, THLE2, HUH7, HEP3B, MHCC97H, SNU387 and HEP1-6 cells were obtained from the Cell Bank of the Chinese Academy of Sciences and cultured in high-glucose DMEM supplemented with 10% fetal bovine serum and 1% penicillin-streptomycin at 37 °C with 5% CO_2_.

Lentiviral vectors were constructed for ITGB5 knockdown and overexpression. Two shRNA sequences targeting ITGB5 were cloned into pLK-0.5, while full-length human or murine ITGB5 coding sequences were inserted into lentiviral overexpression vectors. Lentiviruses were packaged in HEK293T cells using pSPAX2 and pMD2G helper plasmids. Stable cell lines were selected with puromycin and validated by western blotting and flow cytometry.

### Western blotting and densitometric analysis

Total proteins from HCC tissues, paired adjacent tissues, and cultured cells were extracted using RIPA lysis buffer supplemented with protease and phosphatase inhibitors. Samples were lysed on ice for 20 min and centrifuged at 12,000*g* for 15 min at 4 °C. Protein concentrations were determined by BCA assay, and equal amounts of protein (20 μg per lane) were denatured and separated by 8% SDS-PAGE. Proteins were transferred onto 0.45 μm PVDF membranes at 250 mA for 90 min and blocked with 5% non-fat milk in TBST for 1 h at room temperature.

Membranes were incubated overnight at 4 °C with primary antibodies against ITGB5 (4708S, CST, 1:1000) and β-actin (GB15003-100, Servicebio, 1:10000), followed by incubation with HRP-conjugated secondary antibody (GB23303, Servicebio, 1:5000) for 1 h at room temperature. Protein bands were visualized using ECL chemiluminescence reagents, and full uncropped blot images were acquired with an automated imaging system.

Band intensity was quantified using ImageJ-win64 software with a standardized workflow (https://imagej.nih.gov/ij/). Briefly, raw unadjusted blot images were converted to 8 bit grayscale, and background noise was subtracted using a rolling ball algorithm (radius = 50 pixels). The integrated density of each ITGB5 band and its corresponding β-actin band was measured using uniformly sized rectangular frames. Relative ITGB5 expression was calculated as the ratio of ITGB5 integrated density to the internal control density. All experiments were performed in three independent biological replicates. Quantitative data are presented as mean ± SD, and statistical differences were analyzed by unpaired Student’s *t* test (*p* < 0.05).

### Molecular and cellular assays

ITGB5 mRNA and protein expression were measured by quantitative RT-PCR and western blotting. Immunohistochemistry was performed on tissue microarrays and mouse tumor sections using antibodies against ITGB5(28543-1-AP, Proteintech, 1:200), Ki-67, CD8(66868-1-Ig, Proteintech, 1:200) and granzyme B(13588-1-AP, Proteintech, 1:200), as appropriate. H&E staining was used to evaluate tumor morphology and organ toxicity.

Cell proliferation was assessed using CCK-8 and EdU incorporation assays. Clonogenic capacity was evaluated by colony formation assays. Migration and invasion were determined using wound-healing and Matrigel-coated Transwell assays. Apoptosis was measured by Annexin V/PI staining and flow cytometry. Cell-cycle distribution was analyzed by DNA staining and flow cytometry.

### Yeast-display nanobody screening

A yeast-display nanobody library was used to screen ITGB5-binding clones. HEK293T, HUH7 and ITGB5-knockdown SNU387 cells served as negative selection cells, whereas ITGB5-overexpressing HEK293T cells, HUH7 cells and WT SNU387 cells were used for positive selection. After three rounds of cell-based enrichment, plasmids were recovered from yeast, cloned and expressed in HEK293T cells. Supernatants from individual clones were screened by flow cytometry against ITGB5-positive cells. The candidate nanobody Anti-ITGB5-Nb#2 was selected for large-scale production and purified, with purity confirmed by SDS-PAGE and SEC-HPLC.

### Binding characterization

Binding specificity of Anti-ITGB5-Nb#2 was evaluated by immunofluorescence and flow cytometry using cells expressing endogenous or ectopic human or murine ITGB5. Structural models of human (UniProt P18084) and murine (UniProt O70309) ITGB5 extracellular domains (residues 24–719) were obtained from AlphaFold2 database. The anti-ITGB5 VHH monomer structure was predicted by AlphaFold3, and model 0 was selected for docking. Rigid-body protein-protein docking was performed *via* ZDOCK Server 3.0.2, with ITGB5 extracellular domain as receptor and VHH as ligand; no binding-site constraints were added and all other parameters used default settings. The top-ranked human and murine ITGB5-VHH complexes were superimposed against ITGB5 Cα atoms, and intermolecular interactions were analyzed using PLIP v3.0.0 (32).

SPR was performed on a Biacore system using SNU387 cells immobilized on the sensor chip. Serial concentrations of Anti-ITGB5-Nb#2 were injected, and kinetic parameters were calculated using a 1:1 Langmuir binding model (33).

### Antibody production, purification and quality detection

The recombinant ITGB5 antibody used in this study was customized, synthesized and prepared by Generay Biotech using a mammalian 293 cell expression system. Briefly, endotoxin-free plasmids (<1 EU/ml) were transfected into 293 cells, which were cultured at 37 °C with 8% CO_2_ for five days. The cell culture supernatant was harvested by centrifugation at 8000 rpm for 10 min for antibody purification.

Antibody purification was performed using AmMag Protein A Magnetic Beads. After 1 h of bead-supernatant incubation, the beads were thoroughly washed with PBS. The target antibody was eluted with 0.1 M Glycine-HCl (pH 3.5), and each elution fraction was immediately neutralized with 1 M Tris-HCl (pH 8.0). Qualified eluents were pooled and dialyzed for subsequent experiments.

The purified antibody was strictly validated *via* multiple quality-control assays. SDS-PAGE under reducing and non-reducing conditions was used to assess antibody purity. Endotoxin content was detected *via* the Limulus amebocyte lysate gel clot method (sensitivity: 0.25 EU/ml). Antibody homogeneity and monomer purity were analyzed by SEC-HPLC using a BioCore SEC-300 column (5 μm, 4.6 × 150 mm, NanoChrom). The detection conditions were set as follows: mobile phase containing 100 mM PB, 100 mM NaCl and 10% IPA (pH 6.7), flow rate of 0.35 ml/min, sample loading of approximately 10 μg, and detection wavelength of 280 nm.

### Mouse models and treatment

For subcutaneous xenograft experiments, HCC cells were injected into the right axilla of BALB/c nude or C57BL/6 mice. Tumor size and body weight were measured every 3 days, and tumor volume was calculated as 0.5 × length × width^2^. For orthotopic HCC modeling, HUH7-OE cells were injected beneath the liver capsule of BALB/c nude mice. Ten days after tumor implantation, mice received Anti-ITGB5-Nb#2 intravenously at 200 μg per mouse every five days for five doses. Tumor growth was monitored by *in vivo* imaging and magnetic resonance imaging. All animal procedures were approved by the Animal Ethics Committee of the Second Hospital of Lanzhou University (No. D2024-633).

### Single-cell RNA sequencing

Tumor tissues from antibody-treated and untreated control immunocompetent mice were harvested and enzymatically digested with collagenase IV and DNase I to prepare single-cell suspensions. After filtering, red blood cell lysis and viability assessment, three biological replicates were prepared for each group to construct six single-cell libraries. Library construction and sequencing were performed by LC-Bio Technology on the 10 × Genomics Chromium platform, and raw data were analyzed on LC-Bio’s official cloud platform.

Raw sequencing data underwent preprocessing, quality control filtering, log normalization, PCA and UMAP dimensionality reduction, unsupervised clustering and cell type annotation using R (https://www.r‑project.org/) packages Seurat and SingleR (34, 35). We identified major cell subsets including tumor cells, T cells, NK cells, macrophages, dendritic cells and B cells. NK and T cell subclusters were re-clustered to evaluate cytotoxic and activation gene signatures.

To systematically characterize treatment-induced intratumoral immune remodeling, we performed a series of downstream analyses: differential gene expression analysis, GO and KEGG functional enrichment, GSEA (36), pseudotime trajectory analysis, CytoTRACE differentiation potential evaluation (37), CellChat intercellular communication analysis, and pySCENIC transcription factor regulon analysis (38, 39).

### Statistical analysis

Data are presented as mean ± SEM unless otherwise indicated. Statistical analyses were performed using GraphPad Prism (https://www.graphpad.com/) and R. Two-group comparisons were conducted using Student’s *t* test or Wilcoxon rank-sum test, as appropriate. Multiple-group comparisons were performed using one-way or two-way ANOVA with correction for multiple testing. Survival curves were compared using the log-rank test. Correlations were assessed using Pearson or Spearman analysis. *p* < 0.05 was considered statistically significant.

## Consent for publication

All authors consent to publication of this work and confirm that the manuscript is original, has not been published previously, and is not under consideration elsewhere.

## Data availability

Public datasets used in this study, including TCGA-LIHC, ICGC, TIMER2.0 and the Human Protein Atlas, are available from their respective repositories. The clinical specimens, raw experimental data, single-cell sequencing data and analysis code are available from the corresponding author upon reasonable request, subject to a data use agreement for academic purposes only. The nucleotide sequence of the ITGB5-targeting nanobody has been submitted for patent protection and is therefore not publicly disclosed.

## Supporting information

This article contains supporting information.

## Conflict of interest

The authors declare that they have no conflicts of interest with the contents of this article.

## Figures and Tables

**Fig. S5:**