# Supplementary material for: A high-affinity anti-ITGB5 nanobody for hepatocellular carcinoma: Antitumor efficacy and tumor microenvironment reprogramming
Source: J Biol Chem. 2026 Aug 11;302(10):113423. doi: 10.1016/j.jbc.2026.113423 (PMC13570297; doi:10.1016/j.jbc.2026.113423)
Supplement: Supplementary file 1 [file mmc1.docx]

**Fig.s1 Correlation of *ITGB5* expression with clinical characteristics and related molecules, and functional enrichment analysis of differentially expressed genes**

(A) Heatmap displaying the correlation pattern of *ITGB5* expression with multiple clinical pathological parameters of HCC patients；(B) Global correlation heatmap of *ITGB5* with representative immune-related genes；(C)Heatmap of *ITGB5* expression correlated with immune checkpoints；(D,E)Correlation analysis using the TIMER2.0 database: correlated with (D) CD58 and (E) TGFB1；(F) GO functional enrichment analysis of *ITGB5*-related DEGs；(G-K)KEGG (G) and GSEA pathway enrichment analysis: FC_GAMMA_R_MEDIATED_PHAGOCYTOSIS(H),NOTCH_SIGNALING_PATHWAY(I),VEGF_SIGNALING_PATHWAY(J), and ERBB_SIGNALING_PATHWAY (K).

**Fig.s2 Validation of ITGB5 expression in clinical HCC samples and primary screening of nanobodies**

(A)Quantitative WB results of ITGB5 expression in 6 pairs of tumor and adjacent normal tissues from HCC patients；(B)Representative immunohistochemical staining of ITGB5 in 140 pairs of tumor and adjacent normal tissues；(C-F)Flow cytometric screening of antibodies. A total of 32 single clones were selected, among which 6 antibodies exhibited high positive rates；（G）CCK-8 assay for SNU387 cell proliferation: Only the Anti-ITGB5 (Nb)#2 antibody showed inhibitory effects on SNU387 cells (*P* < 0.001). All quantitative data are presented as mean ± SD. ***P* < 0.01, ****P* < 0.001, ns, not significant (*P*> 0.05).

**Fig.s3 In vitro antitumor effects of Anti-ITGB5 (Nb)#2**

(A) Dose-effect evaluation of Anti-ITGB5 (Nb)#2. (a) In vitro IC50 determination via CCK-8 proliferation assay. The fitted IC50 value of Nb#2 was 3.09 μg/mL (approximately 206 nM). (b) In vivo dose–response curve of Nb#2 treatment in tumor-bearing mice. Mice were administrated with gradient nanobody doses, and terminal tumor weights were used to calculate tumor growth inhibition (TGI, %). The curve was fitted using a four-parameter logistic model. Each dot represents an individual replicate (n=3), and data are shown as mean ± SD. The in vivo ED50 of Nb#2 was determined as 8.15 mg/kg.;(B,C) Colony formation assays assessing the long-term proliferative inhibitory effect of Nb#2: In SNU387 (B) and HEP1-6-OE (mITGB5) (C) cells；(D,E) Quantitative analysis of colony formation: In SNU387 (D) and HEP1-6-OE (mITGB5) (E) cells；(F-G) EdU incorporation assays detecting cell proliferation activity after Nb#2 treatment: In SNU387 (F) and HEP1-6-OE (mITGB5) (G) cells；(H,I)Quantitative analysis of EdU staining:In SNU387 (H) and HEP1-6-OE (mITGB5) (I) cells；(J,K) Wound-healing assays evaluating cell migratory capacity following Nb#2 intervention: In SNU387 (J) and HEP1-6-OE (mITGB5) (K) cells；(L,M)Quantitative analysis of wound-healing assay: In SNU387 (L) and HEP1-6-OE (mITGB5) (M) cells. All quantitative data are presented as mean ± SD. ***P* < 0.01, ****P*< 0.001, *****P* < 0.0001, ns, no significant difference (*P* > 0.05).

**Fig.s4 Quality control, clustering, gene heatmap and KEGG analysis of differentially expressed genes from single-cell RNA sequencing**

(A,B) Single-cell transcriptomic data quality control before and after rigorous filtering. Pre-filtered raw data distribution (A) and high-quality data distribution after removing low-quality cells and doublets (B) were displayed to ensure the reliability of subsequent bioinformatic analysis；(C) Statistical quantification and comparison of the proportional distribution of distinct cell subsets in control and Anti-ITGB5 (Nb)#2-treated tumor tissues；(D) Heatmap visualization of the top 10 significantly differentially expressed marker genes across all identified cell clusters, exhibiting specific gene expression patterns of each cell population；(E) UMAP dimensionality reduction plot showing precise cell type annotation of all valid single cells；(F) Sub-clustering analysis of NK cell populations to resolve distinct NK cell subsets with different transcriptional characteristics；(G)Heatmap of differentially expressed genes in NK cell subsets；(H)KEGG pathway enrichment analysis of differentially expressed genes; (I)Volcano plot of differentially expressed genes between the control and treatment groups.

**Fig.s5 Pseudotime analysis, cell‑cell communication analysis, and transcription factor analysis**

(A) Heatmap showing dynamic expression patterns of the top 50 differentially expressed genes across continuous single‑cell pseudotime trajectories; (B) Cell differentiation potential of distinct cell subsets predicted and quantified using the CytoTRACE algorithm；(C) Statistical quantification of the overall intercellular communication strength among major cell populations in control and Anti‑ITGB5 (Nb)#2‑treated；(D)Differential cell‑cell communication analysis:in the treatment group (red lines) and control group (blue lines)；(E,F)Cell‑cell communication analysis quantifying changes in signaling capacity of cell subsets before and after antibody treatment: control group(E), treatment group(F); (G) Transcription factor regulon activity analysis performed via the SCENIC algorithm. Heatmap exhibiting the differential activity patterns of core transcription factor regulons across cell clusters; (H-J) UMAP visualization of transcription factor AUC distribution. Core transcription factors including Foxa2 (H), Sox9 (I), and Pbx1 (J) are highly and specifically activated in tumor cells (Cluster 8) in the control group.
